# A bromodomain-independent mechanism of gene regulation by the BET inhibitor JQ1: direct activation of nuclear receptor PXR

**DOI:** 10.1093/nar/gkad1175

**Published:** 2023-12-12

**Authors:** Andrew D Huber, Shyaron Poudel, Jing Wu, Darcie J Miller, Wenwei Lin, Lei Yang, Monicah N Bwayi, Mary Ashley Rimmer, Rebecca R Florke Gee, Jayaraman Seetharaman, Sergio C Chai, Taosheng Chen

**Affiliations:** Department of Chemical Biology and Therapeutics, St. Jude Children's Research Hospital, 262 Danny Thomas Place, Memphis, TN 38105, USA; Department of Chemical Biology and Therapeutics, St. Jude Children's Research Hospital, 262 Danny Thomas Place, Memphis, TN 38105, USA; Department of Chemical Biology and Therapeutics, St. Jude Children's Research Hospital, 262 Danny Thomas Place, Memphis, TN 38105, USA; Department of Structural Biology, St. Jude Children's Research Hospital, 262 Danny Thomas Place, Memphis, TN 38105, USA; Department of Chemical Biology and Therapeutics, St. Jude Children's Research Hospital, 262 Danny Thomas Place, Memphis, TN 38105, USA; Department of Chemical Biology and Therapeutics, St. Jude Children's Research Hospital, 262 Danny Thomas Place, Memphis, TN 38105, USA; Department of Chemical Biology and Therapeutics, St. Jude Children's Research Hospital, 262 Danny Thomas Place, Memphis, TN 38105, USA; Department of Chemical Biology and Therapeutics, St. Jude Children's Research Hospital, 262 Danny Thomas Place, Memphis, TN 38105, USA; Department of Chemical Biology and Therapeutics, St. Jude Children's Research Hospital, 262 Danny Thomas Place, Memphis, TN 38105, USA; Graduate School of Biomedical Sciences, St. Jude Children's Research Hospital, 262 Danny Thomas Place, Memphis, TN 38105, USA; Department of Structural Biology, St. Jude Children's Research Hospital, 262 Danny Thomas Place, Memphis, TN 38105, USA; Department of Chemical Biology and Therapeutics, St. Jude Children's Research Hospital, 262 Danny Thomas Place, Memphis, TN 38105, USA; Department of Chemical Biology and Therapeutics, St. Jude Children's Research Hospital, 262 Danny Thomas Place, Memphis, TN 38105, USA

## Abstract

Bromodomain and extraterminal (BET) proteins are extensively studied in multiple pathologies, including cancer. BET proteins modulate transcription of various genes, including those synonymous with cancer, such as *MYC*. Thus, BET inhibitors are a major area of drug development efforts. (+)-JQ1 (JQ1) is the prototype inhibitor and is a common tool to probe BET functions. While showing therapeutic promise, JQ1 is not clinically usable, partly due to metabolic instability. Here, we show that JQ1 and the BET-inactive (−)-JQ1 are agonists of pregnane X receptor (PXR), a nuclear receptor that transcriptionally regulates genes encoding drug-metabolizing enzymes such as CYP3A4, which was previously shown to oxidize JQ1. A PXR-JQ1 co-crystal structure identified JQ1′s *tert*-butyl moiety as a PXR anchor and explains binding by (−)-JQ1. Analogs differing at the *tert*-butyl lost PXR binding, validating our structural findings. Evaluation in liver cell models revealed both PXR-dependent and PXR-independent modulation of CYP3A4 expression by BET inhibitors. We have characterized a non-BET JQ1 target, a mechanism of physiological JQ1 instability, a biological function of (−)-JQ1, and BET-dependent transcriptional regulation of drug metabolism genes.

## Graphical Abstract

**Graphical Abstract:**
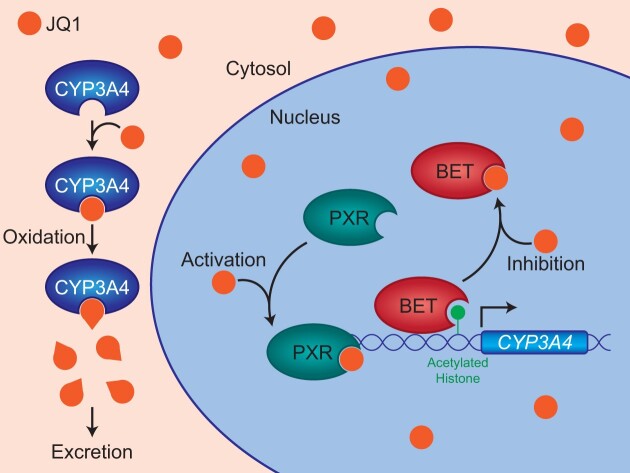

## Introduction

Bromodomains are approximately 110 amino acid protein domains that recognize acetylated lysines in histones and other proteins (1). Bromodomain-containing proteins (BRDs) are large multidomain proteins associated with diverse nuclear roles, including chromatin remodeling, transcriptional control, and methyl or acetyltransferase activities (2,3), and sequence analysis has identified 46 diverse human proteins that contain a total of 61 various bromodomains (3). The bromodomain and extraterminal (BET) family of BRDs, which contains four members (BRD2, BRD3, BRD4 and BRDT), modulates transcription by binding acetylated lysines on histone tails, acting as epigenetic ‘readers’ (4). BET proteins are well known for their roles in multiple cancers. For example, chromosomal rearrangements resulting in fusions of *BRD3* or *BRD4* with nuclear protein in testis (*NUT*) contribute to carcinogenesis in NUT midline carcinomas (NMCs) (5,6), and *BRD4* is an essential gene in luminal breast cancer (7). Furthermore, BET proteins have been revealed as players in multiple pathologies other than cancer, such as renal fibrosis (8) and viral infections (9).

$(+)$ -JQ1 (henceforth referred to as JQ1) was first reported in 2010 as a bromodomain inhibitor exhibiting selectivity for BET family proteins over other BRDs (10), and a close analog, I-BET762, shortly followed (11). BET inhibitors function by competitively binding to the acetyl-lysine binding site of each bromodomain. In addition to the competitive inhibition mechanism, BET inhibitors have been conjugated to E3 ubiquitin ligase ligands to induce degradation of BET proteins, rather than blocking BET interactions with histones. For example, JQ1 linked to thalidomide (dBET1) or VH032 (MZ1) recruits CRL4^CRBN^ or CRL2^VHL^, respectively, resulting in subsequent ubiquitination and proteasomal degradation of BET proteins (12,13). JQ1 is a shining example of a successful chemical biology campaign. Since its discovery, JQ1 has been used extensively as a research tool and prototype for drug development, currently appearing in >1000 PubMed articles. JQ1 and its derivatives have been instrumental in studying the roles of BET proteins in transcription and enhancer function and have allowed rapid progress in the BET field compared to other BRDs (14). However, the discovery of JQ1 was still a catalyst to the entire bromodomain field. In addition to accelerating basic science efforts in bromodomain biology, countless BET inhibitors have now been synthesized, and approximately 20 compounds have entered clinical trials, exhibiting the great translational interest in BRD targets (15). As of the writing of this article, >50 clinical trials containing BET inhibitors have commenced (www.clinicaltrials.gov). JQ1 and related chemicals have shown preclinical effects on a range of conditions in addition to the originally studied NMCs, including solid tumors and hematological malignancies (15), bacterial infections (16), viral infections (9), pulmonary fibrosis (17), liver fibrosis (18) and cardiac hypertrophy (19).

**Figure 1.**
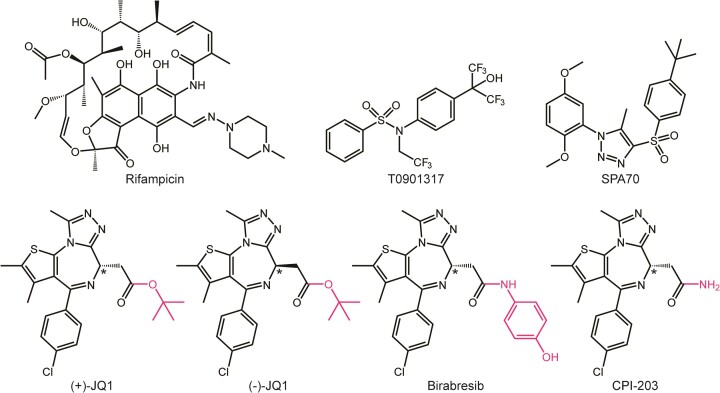
Chemical structures of compounds used in this study. Magenta coloring denotes the chemical changes among the JQ1 analogs. The stereocenter of JQ1 and analogs is indicated by an asterisk.

While BET inhibitors and degraders have significant potential to be part of future therapies, JQ1 itself has limited clinical value. This limitation is partially due to metabolic lability, as JQ1 has been shown to be a substrate for cytochrome P450 3A4 (CYP3A4), a monooxygenase that metabolizes a large proportion of clinical drugs and is responsible for many known drug-drug interactions (20,21). In the current work, we establish that in addition to being a CYP3A4 substrate, JQ1 also induces CYP3A4 expression through activation of the xenobiotic-sensing nuclear receptor pregnane X receptor (PXR). To differentiate between PXR activation and BET inhibition, we show that JQ1 binds directly to the PXR ligand binding domain (LBD), induces recruitment of transcriptional coactivator to PXR, exhibits selectivity for human PXR over mouse PXR (mPXR), and can be blocked by a PXR antagonist. Interestingly, both JQ1 and the BET-inactive enantiomer (−)-JQ1 bind and activate PXR with equal efficiencies, identifying a previously unknown biological function of (−)-JQ1. A co-crystal structure of JQ1-bound PXR LBD shows that the *tert*-butyl group of JQ1 is engaged in stabilizing interactions with an aromatic cage of PXR LBD, and two JQ1 analogs with chemical modifications at the *tert*-butoxy position (birabresib and CPI-203, Figure 1) are weak and inactive, respectively, for PXR binding and activation, validating the importance of *tert*-butyl interactions within the hydrophobic subpocket. The structure and supporting molecular dynamics (MD) simulations reveal conformational flexibility of JQ1 in the PXR ligand binding pocket and clearly indicate how (−)-JQ1 binding is permissible. Importantly, this is the first structurally characterized BET-independent mechanism of transcriptional modulation by a BET inhibitor. Lastly, by assessing CYP3A4 induction in the HepaRG liver cell model, we show that BET inhibitors regulate CYP3A4 transcription in a complex manner that includes both PXR activation and BET inhibition. In summation, our work has identified (i) a non-BET target of JQ1, (ii) a mechanism of physiological JQ1 instability, (iii) a biological function of (−)-JQ1 and (iv) a BET-dependent regulatory pathway for transcription of drug metabolism genes.

## Materials and Methods

### Cell culture

HepG2/C3A cells were obtained from the American Type Culture Collection (ATCC, CRL-10741) and maintained in Eagle's Minimum Essential Medium (ATCC) with 10% FBS (HyClone). HepaRG 5F parental cells were purchased from Sigma-Aldrich and maintained with the HCM Hepatocyte Culture Medium BulletKit (Lonza). Cells were incubated in a humidified atmosphere at 37°C with 5% CO_2_ and routinely verified to be mycoplasma free by using the MycoProbe Mycoplasma Detection Kit (R&D Systems). Cell counts were obtained with a Countess II Automated Cell Counter using trypan blue staining.

### Compounds, reagents and antibodies

DMSO, rifampicin, T0901317, and pregnenolone-16α-carbonitrile (PCN) were purchased from Sigma-Aldrich. SPA70 was obtained from WuXi AppTec (22). (+)-JQ1, (−)-JQ1, birabresib, and CPI-203 were purchased from MedChemExpress. All compound purities were determined to be >95% by the manufacturers. LanthaScreen Terbium (Tb)-anti–glutathione *S*-transferase (GST, cat. # PV3550) and GST-PXR LBD protein (cat. # PV4841) were purchased from Thermo Fisher Scientific. Unfused GST protein was purchased from Abcam (cat. # ab70456). FLAG-RXRα protein was purchased from Active Motif (cat. # 81782). Mouse anti-FLAG M2-FITC antibody was purchased from Sigma-Aldrich (cat. # F4049). Unlabeled and FAM-labeled SRC-1 peptides (N-CPSSHSSLTERHKILHRLLQEGSPS-C) were prepared by the Hartwell Center Macromolecular Synthesis Section at St. Jude Children's Research Hospital (23). FAM-labeled NCoR peptide (N-DPASNLGLEDIIRKALMGSFDDK-C) was also prepared by the Hartwell Center Macromolecular Synthesis Section at St. Jude Children's Research Hospital (22). BODIPY FL vindoline was synthesized as previously reported (24). Mouse anti-PXR (H-11) antibody was purchased from Santa Cruz Biotechnology (cat. # sc-48340). Rabbit anti-β-actin antibody was purchased from Cell Signaling Technology (cat. # 4967L). Goat anti-mouse IRDye 800CW (cat. # 926–32210) and goat anti-rabbit IRDye 680LT (cat. # 926–68021) secondary antibodies were purchased from LI-COR.

### Plasmids

Construction of the pcDNA3-FLAG-PXR expression plasmid was described previously using pcDNA3 from Thermo Fisher Scientific (25). Construction of pGL3-CYP3A4-luc containing firefly luciferase under the control of a PXR-responsive *CYP3A4* promoter has also been described (25,26). The pRL-CMV vector containing *Renilla* luciferase under the control of the CMV promoter was purchased from Promega. The pGL4.54[luc2/TK], pGL4.53[luc2/PGK] and pGL4.13[luc2/SV40] vectors containing firefly luciferase under the control of TK, PGK, and SV40 promoters, respectively, were purchased from Promega. To generate pGL4.53-PGK-PXR containing PGK-driven PXR, pGL4.53[luc2/PGK] was digested with NcoI and XbaI, and the vector band was gel purified. PXR was amplified from pcDNA3-FLAG-PXR using primers 5′-GGTACTGTTGGTAAAGCCACCATGGAGGTGAGACCCAAAGAAAG-3′ and 5′-AGCGGCCGGCCGCCCCGACTCTAGATCAGCTACCTGTGATGCC-3′ and ligated to the digested vector with NEBuilder HiFi DNA Assembly Master Mix (New England BioLabs). The mPXR expression vector (pCMV6-mPXR Kana-R) was purchased from OriGene Technologies. The pETDuet-1 vector containing His-PXR LBD (residues 130–434) and untagged mouse SRC-1 (residues 623–710) was described previously (22,27,28). The Q5 Site-Directed Mutagenesis Kit (New England BioLabs) was used to generate pcDNA3-SmBiT-PXR containing full-length PXR with an N-terminal SmBiT peptide in place of FLAG with pcDNA3-FLAG-PXR template and 5′-CTGTTCGAGGAGATTCTGGGACCAAGCTTGGTACCGAGCTCG-3′ and 5′-CCGGTAGCCGGTCACCATGGTGGCAGCTTGGGTCTCCC-3′ primers. The Q5 Site-Directed Mutagenesis Kit was then used to generate pcDNA3-SmBiT-PXR LBD (residues 139–434) with pcDNA3-SmBiT-PXR template and 5′-GGAGTGCAGGGGCTGACAGAGGAGCAGCGG-3′ and 5′-GTTAAGGGCGAATTCCAGCACACTGGCGGCCGTTAC-3′ primers. The pBIND-SRC-1 vector containing the nuclear receptor interaction domain of steroid receptor coactivator-1 (SRC-1, amino acids 621–765) has been described previously (29). The SRC-1 fragment was amplified from this vector using 5′-TGCTGGAATTCGCCCTTAACGATGGAGACAGTAAATACTCTCAAACCAGTC-3′ and 5′-AGATGCATGCTCGAGGATCCTCAGTTTGGAGTTGATCTTAAATCTTTCTCATCTTTATC-3′ primers. Primers 5′-TCTTCGAGTGTGAAGACCATGGTGGCAGCTTGGGTCTCCCTATAG-3′ and 5′-GGATCCTCGAGCATGCATCTAGAGGGCCCTATTC-3′ were used to amplify the pcDNA3-FLAG-PXR backbone, removing the FLAG-PXR open reading frame. To generate pcDNA3-LgBiT-SRC-1, NEBuilder HiFi DNA Assembly Master Mix was used to assemble the SRC-1 and pcDNA3 amplicons with a gBlock (Integrated DNA Technologies) containing LgBiT and linker sequences (5′-ATGGTCTTCACACTCGAAGATTTCGTTGGGGACTGGGAACAGACAGCCGCCTACAACCTGGACCAAGTCCTTGAACAGGGAGGTGTGTCCAGTTTGCTGCAGAATCTCGCCGTGTCCGTAACTCCGATCCAAAGGATTGTCCGGAGCGGTGAAAATGCCCTGAAGATCGACATCCATGTCATCATCCCGTATGAAGGTCTGAGCGCCGACCAAATGGCCCAGATCGAAGAGGTGTTTAAGGTGGTGTACCCTGTGGATGATCATCACTTTAAGGTGATCCTGCCCTATGGCACACTGGTAATCGACGGGGTTACGCCGAACATGCTGAACTATTTCGGACGGCCGTATGAAGGCATCGCCGTGTTCGACGGCAAAAAGATCACTGTAACAGGGACCCTGTGGAACGGCAACAAAATTATCGACGAGCGCCTGATCACCCCCGACGGCTCCATGCTGTTCCGAGTAACCATCAACAGTGGACCAAGCTTGGTACCGAGCTCGGATCCACTAGTAACGGCCGCCAGTGTGCTGGAATTCGCCCTTAAC-3′). The plasmids pET-His-MBP-TEV-His-LIC-hCAR1-LBD (a gift from Dr. Elias Fernandez, University of Tennessee-Knoxville) and pcDNA3.1(+)-RXRα (obtained from the cDNA Resource Center, cdna.org) were not used directly in this study but were used to generate pET-His-MBP-TEV-RXRα for bacterial expression of full-length RXRα. The LIC cleavage sequence and second His tag of pET-His-MBP-TEV-His-LIC-hCAR1-LBD were removed with the QuikChange II Site-Directed Mutagenesis Kit (Agilent Technologies) using primers 5′-ATGCCTGTGCAACTGAGTAAGGA-3′ and 5′-GGATTGGAAGTACAGGTTTTCCT-3′ to generate pET-His-MBP-TEV-hCAR1-LBD. This vector was digested with NdeI and XhoI and gel purified to remove the hCAR1 LBD. The RXRα coding sequence was amplified from pcDNA3.1(+)-RXRα with primers 5′-TCCCATATGGACACCAAACATTTCCTGCCGC-3′ and 5′-TGCTCGAGCTAAGTCATTTGGTGCGGCGCCT-3′, digested with NdeI and XhoI, and ligated to the digested vector with T4 DNA ligase.

### PXR transactivation assays

Assays were performed similarly as previously described with minor modifications (30–32). HepG2 cells (7.5 × 10^5^/well) were plated in six-well tissue culture-treated plates. The following day, cells were co-transfected with pGL3-CYP3A4-luc (2 μg/well), pRL-CMV (200 ng/well) and 100 ng/well of either empty vector (pcDNA3), pcDNA3-FLAG-PXR, pCMV6-mPXR or pGL4.53-PGK-PXR using Lipofectamine 3000 (Thermo Fisher Scientific). Twenty-four hours after transfection, cells were trypsinized and suspended in phenol red-free DMEM (Thermo Fisher Scientific) supplemented with 5% charcoal/dextran-treated FBS (HyClone), and 1 × 10^4^ cells/well in 25 μl media were added to white 384-well plates. An Echo 650 Acoustic Liquid Handler (Labcyte Inc.) was used to dispense 125 nl/well of DMSO or stock compounds, resulting in 0.5% DMSO and the indicated concentrations of chemicals. After 24 h, the Dual-Glo Luciferase Assay System (Promega) and EnVision microplate reader (PerkinElmer) were used to measure firefly and *Renilla* luciferase activities. CMV-driven *Renilla* luciferase was used as an indicator of general transcriptional modulation by the test compounds and is plotted as fold change (FC) relative to DMSO controls. Assays utilizing pGL4.54[luc2/TK], pGL4.53[luc2/PGK], or pGL4.13[luc2/SV40] were similarly performed and normalized. For the PXR-responsive CYP3A4-luciferase reporter, the measured relative light units (RLU) were normalized for each well using equation (1),


\begin{eqnarray*}RLU\ \left( \% \right) = 100 \times \left( {\frac{{\left( {RL{U}_{Chemical} - RL{U}_{DMSO}} \right)}}{{\left( {RL{U}_{T0901317} - RL{U}_{DMSO}} \right)}}} \right)\end{eqnarray*}

RLU_T0901317_ represents the measured RLU from wells containing 1 μM T0901317. For mPXR assays, 5 μM PCN was used in place of T0901317.

### Cytotoxicity assay

HepG2 cells (1 × 10^4^/well in 25 μl phenol red-free DMEM supplemented with 5% charcoal/dextran-treated FBS) were plated and treated as in the transactivation assays. After 24 h compound treatment, the CellTiter-Glo Luminescent Cell Viability Assay (Promega) and an EnVision microplate reader were used to assess cytotoxicity. DMSO wells with cells served as negative controls, and wells without cells served as positive controls. The averaged luminescence signal of the wells without cells was subtracted from the luminescence signals of all other wells, and the background subtracted values were normalized to DMSO-treated wells containing cells to obtain percent cell viability.

### Time-resolved fluorescence resonance energy transfer (TR-FRET) PXR LBD SRC-1 and NCoR recruitment assays

The TR-FRET PXR LBD SRC-1 recruitment assay measures PXR LBD interaction with a fluorescently labeled SRC-1 peptide (Supplementary Figure S1) and was performed as previously described with minor modifications (23). The assay buffer composition was 50 mM Tris (pH 7.5), 20 mM MgCl_2_, 0.1 mg/ml bovine serum albumin, and 0.05 mM dithiothreitol. FAM-SRC-1 peptide solution (15 μl/well, 133.3 nM in assay buffer) was dispensed into 384-well black low-volume assay plates. An Echo 650 Acoustic Liquid Handler then dispensed 60 nl/well of compound stocks or DMSO. Lastly, 5 μl/well of 20 nM Tb-anti-GST antibody and 20 nM GST-PXR LBD protein in assay buffer was added. The final assay volume per well was 20 μl, and the final concentrations for the assay components were: 100 nM FAM-SRC-1 peptide, 5 nM Tb-anti-GST, 5 nM GST-PXR LBD protein and 0.3% DMSO. DMSO alone (0.3%) and 10 μM T0901317 (diluted from 60 nl of 3.33 mM stock to a 20-μl assay volume) were included in each plate to serve as negative and positive controls, respectively. The plates were shaken at 900 rpm (80 × *g*) on an IKA MTS 2/4 digital microtiter shaker for 1 min then centrifuged at 1000 rpm (201 × *g*) for 30 s in an Eppendorf 5810 centrifuge equipped with an A-4-62 swing-bucket rotor. The plates were protected from light exposure and incubated for 1 h at room temperature (RT). After incubation, the TR-FRET signal from each well was collected with a PHERAstar FS Microplate Reader (BMG Labtech). The measured relative fluorescence units (RFU) were normalized for each well using equation (2),


\begin{eqnarray*}Signal = \frac{{RFU\ at\ 520\ nm}}{{RFU\ at\ 490\ nm}}\end{eqnarray*}

and equation (3),


\begin{eqnarray*}TR - FRET\ Signal\ \left( \% \right) &=& 100 \\ &&\times \frac{{\left( {Signa{l}_{Chemical} - Signa{l}_{DMSO}} \right)}}{{\left( {Signa{l}_{T0901317} - Signa{l}_{DMSO}} \right)}}\end{eqnarray*}

The TR-FRET PXR LBD NCoR recruitment assay was similarly performed, but with 500 nM FAM-NCoR peptide. Data were normalized using equations (2) and (3) with 10 μM SPA70 in place of T0901317 as the control. For the NCoR release assay, all wells contained 1 μM SPA70 to induce PXR LBD interaction with NCoR, and dose responses of agonists were used to disrupt the PXR LBD/SPA70/NCoR complex. In the release assay, 1 μM SPA70 alone served as the 100% control, and DMSO alone served as the 0% control. NCoR release assay wells contained 0.4% DMSO due to the two-compound addition.

### TR-FRET PXR LBD RXRα heterodimerization assays

The TR-FRET PXR LBD RXRα heterodimerization assay measures PXR LBD interaction with full-length RXRα (Supplementary Figure S2). The assay buffer composition was 50 mM Tris (pH 7.5), 20 mM MgCl_2_, 0.1 mg/ml bovine serum albumin, and 0.05 mM dithiothreitol. Assays were performed in two stages: (i) assay development and validation and (ii) assays in the presence of PXR ligands. For development and validation, two-fold dilutions of FLAG-RXRα were placed in 384-well black low-volume assay plates (5 μl/well with the highest concentration being 600 nM). Then, 5 μl/well of assay buffer or assay buffer containing 3 μM His-PXR LBD (purified as described in the crystallization subsection below) or 3 μM untagged RXRα (purified as described below) was dispensed into wells. Finally, 5 μl/well of assay buffer containing 600 nM FITC-anti-FLAG, 9 nM Tb-anti-GST and 9 nM GST-PXR LBD or 9 nM unfused GST was dispensed into wells. Plates were shaken, centrifuged, incubated, and measured as above. Final concentrations in the wells were 3 nM GST-PXR LBD or unfused GST, 3 nM Tb-anti-GST, 200 nM FITC-anti-FLAG and 2-fold dilutions of FLAG-RXRα beginning at 200 nM. Indicated wells contained 1 μM His-PXR LBD or 1 μM untagged RXRα to compete with signal generation as a validation step. Wells containing 100 nM FLAG-RXRα and wells without FLAG-RXRα served as positive and negative controls, respectively. The measured RFU were normalized for each well using equations (2) and (4),


\begin{eqnarray*}&&TR - FRET\ Signal\ \left( \% \right) = 100 \\ &&\times \frac{{\left( {Signa{l}_{Well} - Signa{l}_{0\ nM\ FLAG - {\mathrm{RXR\alpha }}}} \right)}}{{\left( {Signa{l}_{100{\mathrm{\ nM\ FLAG}} - {\mathrm{RXR\alpha }}} - Signa{l}_{0\ nM\ FLAG - {\mathrm{RXR\alpha }}}} \right)}}\end{eqnarray*}

For assays with PXR ligands, two-fold dilutions of FLAG-RXRα were placed in the wells (5 μl/well with the highest concentration being 600 nM). Then, 5 μl/well of assay buffer containing 1.5% DMSO with or without 30 μM compound was dispensed into wells. Finally, 5 μl/well of assay buffer containing 600 nM FITC-anti-FLAG, 9 nM Tb-anti-GST, and 9 nM GST-PXR LBD was dispensed into wells. Plates were shaken, centrifuged, incubated, and measured as above. Final concentrations in the wells were 3 nM GST-PXR LBD, 3 nM Tb-anti-GST, 200 nM FITC-anti-FLAG, two-fold dilutions of FLAG-RXRα beginning at 200 nM, 0.5% DMSO, and 10 μM compound as indicated. DMSO-treated wells containing 100 nM FLAG-RXRα or 0 nM FLAG-RXRα served as positive and negative controls, respectively. The measured RFU were normalized for each well using equations (2) and (4).

Untagged full-length RXRα was purified by the Protein Production Facility at St. Jude Children's Research Hospital as follows. The pET-His-MBP-TEV-RXRα plasmid was transformed into *Escherichia coli* BL21(DE3) (Sigma-Aldrich), grown in LB media containing 30 μg/ml kanamycin to an OD_600_ of 0.6, and induced with 0.2 mM isopropyl-β-d-thiogalactopyranoside (IPTG) for 10 h at 20°C. Cells were collected by centrifugation at 4000 × *g*, resuspended in lysis buffer [20 mM Tris–HCl (pH 8.0), 300 mM NaCl, 20 mM imidazole, 1 mM Tris(2-carboxyethyl)phosphine hydrochloride (TCEP)] supplemented with Roche cOmplete, Mini, EDTA-free Protease Inhibitor Cocktail, lysed with a microfluidizer, and centrifuged at 16 000 × *g* for 1 h at 4°C. The supernatant was applied to nickel agarose beads (Gold Biotechnology), the beads were washed with lysis buffer containing 50 mM imidazole, and bound proteins were eluted with lysis buffer containing 250 mM imidazole. His-MBP was cleaved from RXRα with TEV protease, and the mix was dialyzed into buffer A [25 mM Bis–Tris (pH 6.5), 100 mM NaCl, 1 mM TCEP] overnight at 4°C, applied to a HiTrap SP HP cation exchange chromatography column (Cytiva), and eluted with a linear gradient of buffer A to buffer B [25 mM Bis–Tris (pH 6.5), 1 M NaCl, 1 mM TCEP]. Fractions containing pure untagged RXRα were pooled, exchanged to storage buffer [PBS (pH 7.4), 200 mM NaCl, 10% glycerol, 1 mM 4-(2-aminoethyl)benzenesulfonyl fluoride hydrochloride (AEBSF)] with PD-10 desalting columns packed with Sephadex G-25 (Cytiva), and stored at -80°C.

### TR-FRET PXR LBD ligand binding assay

The TR-FRET PXR LBD ligand binding assay measures binding of PXR ligands by competition with a fluorescently labeled ligand (BODIPY FL vindoline) (Supplementary Figure S3) and was performed as previously described with minor modifications (24). The assay buffer composition was 50 mM Tris (pH 7.5), 20 mM MgCl_2_, 0.1 mg/ml bovine serum albumin, and 0.05 mM dithiothreitol. BODIPY FL vindoline (15 μl/well, 133.3 nM in assay buffer) was dispensed into 384-well low-volume black assay plates. An Echo 650 Acoustic Liquid Handler then dispensed 60 nl/well of compound stocks or DMSO. Lastly, 5 μl/well of 20 nM Tb-anti-GST and 20 nM GST-PXR LBD in assay buffer was added. The final concentrations of the assay components in a 20 μl final assay volume per well were: 100 nM BODIPY FL vindoline, 5 nM Tb-anti-GST, 5 nM GST-PXR LBD, and 0.3% DMSO. DMSO alone (0.3%) and 10 μM T0901317 (diluted from 60 nl of 3.33 mM stock to a 20 μl assay volume) were included in each plate to serve as negative and positive controls, respectively. Plates were shaken, centrifuged, incubated, and measured as above. The measured RFU were normalized for each well using equations (2) and (5),


\begin{eqnarray*}&& TR - FRET\ Signal\ \left( \% \right) = 100 \\ &&\times \left( {1 - \frac{{\left( {Signa{l}_{Chemical} - Signa{l}_{T0901317}} \right)}}{{\left( {Signa{l}_{DMSO} - Signa{l}_{T0901317}} \right)}}} \right)\end{eqnarray*}

### Cellular PXR LBD SRC-1 recruitment assay

Nano Luciferase Binary Technology (NanoBiT) (33) was used to assess the interaction of SRC-1 with PXR LBD in HepG2 cells (Supplementary Figure S4). HepG2 cells (7.5 × 10^5^/well) were plated in six-well tissue culture-treated plates. The following day, cells were co-transfected with pcDNA3-SmBiT-PXR LBD (100 ng/well) and pcDNA3-LgBiT-SRC-1 (100 ng/well) using Lipofectamine 3000. Empty vector (pcDNA3, 800 ng/well) was used as carrier DNA due to the low amounts of expression plasmids used. Twenty-four hours after transfection, cells were trypsinized and suspended in phenol red-free DMEM supplemented with 5% charcoal/dextran-treated FBS, and 1 × 10^4^ cells/well in 20 μl media were added to white 384-well plates. After 24 h, 5 μl media containing 2.5% DMSO, 5X Nano-Glo Live Cell Substrate from the Nano-Glo Live Cell Assay System (Promega), and 5× concentration of compound was added, resulting in 0.5% DMSO and either 100 nM T0901317 or 5 μM other compound. The plates were incubated for 30 min at RT, and Nano Luciferase activity was then measured with an EnVision microplate reader.

### His-PXR LBD purification, crystallization and structure determination

PXR LBD was expressed and purified as previously described, with modifications (22,27,28). Codon-optimized sequences for His-tagged PXR LBD (residues 130–434) and untagged mouse SRC-1 (residues 623–710) were cloned into pETDuet-1 (Novagen), which allows co-expression of two genes from separate inducible T7 promoters. The plasmid was transformed into TurboCells Competent *E. coli* BL21(DE3) (Genlantis), grown in terrific broth containing 100 μg/ml ampicillin at 37°C to an OD_600_ of 3–4, and induced overnight at 16°C with 500 μM IPTG. Cells were pelleted by centrifugation at 4000 × *g* and resuspended in lysis buffer [20 mM Tris (pH 7.5), 250 mM NaCl, 5% glycerol, 10 mM imidazole] supplemented with EDTA-free SIGMAFAST protease inhibitor cocktail tablets (Sigma-Aldrich) and 1 mg/ml lysozyme (Thermo Fisher Scientific). The suspension was sonicated and then centrifuged at 20 000 × *g* for 1 h at 4°C, and the supernatant was applied to a 5 ml HisTrap FF column (Cytiva). The column was washed with 50 ml lysis buffer, and bound proteins were eluted with lysis buffer containing 500 mM imidazole. Elution fractions were collected and analyzed by SDS-PAGE for protein amount and purity. Selected fractions were pooled, and a 2:1 molar ratio of SRC-1 peptide was added to stabilize PXR LBD. The PXR LBD/peptide mixture was concentrated to ≤10 ml in a Amicon Ultra-15 centrifugal filter unit with 10 kDa cutoff (Sigma-Aldrich), filtered through a 0.22 μm syringe filter, and loaded onto a HiLoad 26/600 Superdex 200 pg size exclusion column (Cytiva) equilibrated with storage buffer [20 mM Tris (pH 7.8), 250 mM NaCl, 5% (v/v) glycerol, 5 mM DTT, 1 mM EDTA]. Elution fractions were collected and analyzed by SDS-PAGE, pure fractions were pooled, and a 2:1 molar ratio of SRC-1 peptide was again added. The PXR LBD/peptide mixture was concentrated to 4.7 mg/ml, aliquoted, flash frozen in liquid nitrogen, and stored at –80°C.

For crystallization, an aliquot of PXR LBD (4.7 mg/ml) was buffer exchanged to 20 mM Tris (pH 7.8), 200 mM NaCl, 5% (v/v) glycerol, 5 mM DTT, 2.5 mM EDTA using PD-10 desalting columns packed with Sephadex G-25 resin (Cytiva). The exchanged protein eluted at 1.5 mg/ml (41.5 μM). SRC-1 peptide was added to 100 μM, and protein was concentrated to 3.6 mg/ml (100 μM). The protein was mixed with 2 mM JQ1 and incubated for 1 h at 4°C. This mixture contained 2% DMSO from the compound dilution. Hanging drop trays were set with 1 μl protein-ligand complex and 1 μl reservoir solutions containing 50 mM imidazole (pH 6.8–7.8) and 8–14% isopropanol. Crystals grew in various conditions within 3–5 days. The data presented were collected from a single crystal grown for three days in 50 mM imidazole (pH 7) with 8% isopropanol and cryoprotected in a solution containing 50 mM imidazole (pH 7), 1 mM JQ1, 1% DMSO and 40% ethylene glycol.

X-ray diffraction data were collected to a resolution of 2.15 Å at AMX Beamline 17-ID-1 at the National Synchrotron Light Source II at Brookhaven National Laboratory. Frames were processed with XDS (34). The crystal belonged to space group *P*2_1_2_1_2_1_ with two PXR LBD molecules and two SRC-1 peptide molecules in the asymmetric unit. The structure was solved by molecular replacement in Phaser (35) using PDB ID 1NRL as the search model (36). The search model was stripped of solvent and ligand prior to molecular replacement. Iterative cycles of model building and refinement were performed in Coot (37) and Phenix (38). The data collection and refinement statistics are shown in Table 1. All crystallographic figures were made in PyMOL (Schrödinger). The structure is deposited as PDB ID 8F5Y.

**Table 1.** Data collection and model refinement statistics for the JQ1-bound PXR LBD structure (PDB ID 8F5Y)

Data collection		
Resolution range (Å)		32.61–2.15 (2.21–2.15)
Space group		*P* 2_1_ 2_1_ 2_1_
Unit cell dimensions		
	*a*, *b*, *c* (Å)	75.55, 82.56, 103.67
	α, β, γ (°)	90.0, 90.0, 90.0
Wavelength (Å)		0.9201
Unique reflections		36061
Redundancy		6.7 (6.3)
Completeness (%)		99.8 (98.7)
*I*/σ*I*		12.9 (2.3)
*R*_sym_		0.084 (0.797)
CC_1/2_		0.999 (0.829)
**Model refinement**		
*R*_work_/*R*_free_		0.207/0.216
Number of atoms		
	Protein	4354
	Ligand	62
	Water	96
RMSD		
	Bond length (Å)	0.007
	Bond angles (°)	0.933
Ramachandran plot (%)		
	Preferred	98.29
	Outliers	0.00
Clashscore^†^		1.71
MolProbity score^†^		0.93
Average *B*-factor (Å^2^)		49.28
	Protein	48.90
	Ligand	80.72
	Water	46.22

Values from the highest resolution shell are shown in parentheses. †Generated with MolProbity.

### Liquid chromatography tandem mass spectrometry (LC–MS/MS) test of JQ1 stability

An Echo 650 Acoustic Liquid Handler was used to dispense 50 nl/well of 1 mM JQ1 into 96-well assay plates (Corning Inc., one plate per time point, six replicate wells per time point). Crystallization solution (50 μl) was added to each well to yield final concentrations of 1 μM JQ1 and 0.1% DMSO. The crystallization solution contained 25 mM imidazole, 5% isopropanol, 10 mM Tris, 100 mM NaCl, 2.5% (v/v) glycerol, 2.5 mM DTT and 1.25 mM EDTA, and had a final pH of 7.4. This composition matched the final composition of a crystallization drop containing a 1:1 ratio of protein to reservoir solution. To the 0-day plate, 100 μl cold acetonitrile (ACN) containing 400 ng/ml warfarin (Fluka Chemical Corporation) as the internal standard was immediately added to each well. The remaining plates were incubated at 20°C for 1–8 days followed by quenching with 100 μl cold ACN containing 400 ng/ml warfarin. Quenched samples were sealed and stored at -20°C until analysis. On the day of analysis, plates were thawed, shaken at 600 rpm for 10 min, and centrifuged at 2000 × *g* for 10 min. The supernatants (20 μl) were transferred to a new analytical 96-well plate (Corning Inc.) and diluted with 180 μl 50% ACN in Milli-Q water. Chromatographic separation was performed on an ACQUITY UPLC BEH C18 column (1.7 μm, 2.1 mm × 50 mm) (Waters Corporation) using the Triple Quad 6500 System (AB Sciex). The injection volume was 7 μl. The UPLC column was maintained at 60°C. Mobile phase A was 0.1% formic acid in Milli-Q water, and solvent B was 0.1% formic acid in ACN. The flow rate was 0.7 ml/min with a gradient of mobile phase B as follows: 0 min → 0.2 min: 30%; 0.2 min → 1.5 min: 30% → 95%; 1.5 min → 1.8 min: 95%; 1.8 min → 1.95 min: 95% → 30%; 1.95 min → 2 min: 30%. The mass spectrometer was operated in positive ion mode with electrospray ionization. Reaction monitoring parameters were as follows: the pressure of ion source gas 1 = 40 psi, the pressure of ion source gas 2 = 40 psi, the pressure of curtain gas = 30 psi, the pressure of collision gas = 8 psi, ion spray voltage = 5.5 kV, source temperature = 300°C. The multiple reaction monitoring transitions were m/z 457.033 to *m*/*z* 401 for JQ1 and *m*/*z* 309.1 to *m*/*z* 163 for warfarin. The declustering potential, entrance potential, collision energy, and collision cell exit potential were set at 31, 10, 19, and 22 V for JQ1 and 57, 10, 44, and 20 V for warfarin. Data were acquired using Analyst Software (AB Sciex, version 1.6.3) and analyzed using MultiQuant Software (AB Sciex, version 3.0.3).

### LC–MS/MS identification of PXR LBD-bound JQ1

PXR LBD-JQ1 co-crystals were grown as above. Crystals (*n* = 8) were collected and washed in ten successive drops (5 μl each) of crystallization solution (see previous section for components) to remove unbound JQ1. The final washed crystals were combined into 5 μl DMSO. All wash solutions and the final DMSO were collected into 1.5 ml tubes, quenched with 30 μl cold ACN containing 40 ng/ml warfarin as the internal standard, and centrifuged at 16 000 × *g* for 10 min. The supernatants (30 μl each) were transferred to analytical 384-well plates (Corning Inc.) and mixed thoroughly with 30 μl Milli-Q water by pipetting. Finally, 10 μl of each mixture was injected into the LC–MS/MS system and analyzed as above. For the JQ1 standard curve, 10-fold dilutions beginning at 10 μM were made in the crystallization solution and analyzed using the same methods as those used for the test samples. The peak area ratio was calculated by dividing the JQ1 peak area by the internal standard (warfarin) peak area for each injection. A linear regression with 1/*x*^2^ weighting was used to process the data.

### Molecular dynamics (MD) simulations

The structures of PXR LBD bound to JQ1 (chain A) or the second bromodomain of BRD2 bound to JQ1 (PDB ID: 3ONI) (10) were loaded into Coot. Missing loops in PXR LBD were filled using the AlphaFold model of PXR as a guide (39,40), missing sidechains were added, and alternate conformations and solvent atoms were removed. The prepared PDB files were uploaded to the Galaxy web platform, and we used the public server at https://usegalaxy.org for initial MD setup (41). The ligand and protein were split into separate PDB files using the *Search in textfiles* tool. Protein topologies were generated using the *GROMACS initial setup* tool with the AMBER99SB-ILDN force field (42). Hydrogens were added to JQ1 at pH 7.0 using the *Compound conversion* tool, and the *Generate MD topologies for small molecules* tool was used to obtain JQ1 topologies by acpype with the GAFF2 force field (43). The topology and GRO files for the proteins and JQ1 were merged with the *Merge GROMACS topologies* tool, the *GROMACS structure configuration* tool was used to configure a 1.0 nm triclinic simulation box, and the *GROMACS solvation and adding ions* tool was used to solvate the box with the SPC water model and neutralizing ions. The systems were minimized with the *GROMACS energy minimization* tool using default settings for 50 000 steps with an EM tolerance of 1000. The *GROMACS simulation* tool was used to perform four stages of isothermal-isochoric (NVT) equilibration at 50, 100, 200 and 300 K. Default settings were used for each stage with 50 000 steps, a step length of 2 fs, and h-bond constraints. The *GROMACS simulation* tool was then used to perform a single stage of isothermal-isobaric (NPT) equilibration with 50 000 steps, a step length of 2 fs, h-bond constraints, and a temperature of 300 K. The equilibrated systems were downloaded from the Galaxy server, and GROMACS release 2023.2 was used to perform 500 ns NPT simulations with a step length of 2 fs, sampling frequency of 10 ps, and h-bond constraints (44). Simulations were analyzed using GROMACS analysis tools.

### RNA induction in HepaRG

Experiments with HepaRG cells were conducted similarly to previously described (45), with modifications. HepaRG cells were seeded in collagen-coated 24-well plates at 5 × 10^5^ cells/well in HCM. Cells were differentiated for two weeks with HCM containing 2% DMSO. The cells were then treated with the respective chemicals for 24 h in HCM. The final DMSO concentration for the treatment was 0.5%. Total RNA was isolated from cells with Maxwell 16 LEV SimplyRNA Tissue Kits (Promega), and cDNA was generated from 250 ng of RNA with the SuperScript VILO cDNA Synthesis Kit (Thermo Fisher Scientific). Quantitative real-time polymerase chain reaction (RT-qPCR) was conducted with 2 μl of cDNA using TaqMan Fast Advanced Master Mix (Applied Biosystems) in an Applied Biosystems 7500 Fast Real-Time PCR System. TaqMan gene expression assays specific for *CYP3A4* (Hs00604506_m1), *PXR* (Hs01114267_m1), *MYC* (Hs00153408_m1) and *RNA18S* (Hs03928990_g1) were purchased from Thermo Fisher Scientific. Fold induction values were calculated according to the 2^−ΔΔCt^ method, where ΔCt represents the differences in cycle threshold numbers between the target gene and reference gene and ΔΔCt represents the relative change in these differences between the control and treatment groups (46). *RNA18S* was used as the reference gene for relative quantification of all other genes.

### Western blot in HepaRG

HepaRG cells were seeded in collagen-coated 6-well plates at 2 × 10^6^ cells/well in HCM. Cells were differentiated for two weeks with HCM containing 2% DMSO. The cells were then treated with the respective chemicals for 24 h in HCM. The final DMSO concentration for the treatment was 0.5%. Cells were trypsinized, pelleted by centrifugation, washed with PBS, and lysed in radioimmunoprecipitation assay (RIPA) buffer [50 mM Tris (pH 8.0), 150 mM NaCl, 1% NP-40, 0.5% sodium deoxycholate, 0.1% SDS]. Protein in the lysate was quantified with the Pierce BCA Protein Assay Kit (Thermo Fisher Scientific), and 80 μg was loaded with NuPAGE LDS Sample Buffer (Thermo Fisher Scientific) into NuPAGE 4–12% Bis–Tris gels (Thermo Fisher Scientific). Separated proteins were transferred to nitrocellulose membranes using the iBlot 2 Dry Blotting System (Thermo Fisher Scientific). Membranes were blocked with TBST [50 mM Tris (pH 7.4), 150 mM NaCl, 0.1% Tween 20] containing 5% milk for 1 h at RT. Mouse anti-PXR (1:1000 dilution) and rabbit anti-β-actin (1:2000 dilution) were bound overnight at 4°C in TBST containing 5% milk. Membranes were washed with TBST three times for 10 min each, and anti-mouse or anti-rabbit IRDye antibodies (1:10 000 dilution) were added in TBST containing 5% milk for 1 h at RT. Membranes were washed as above and imaged with an Odyssey CLx imaging system (LI-COR).

### Plotting and statistical analyses

All plots were made in GraphPad Prism 9. Results are expressed as the mean ± standard deviation from at least three independent experiments, and basic dose response curves were fitted as needed. Significance was assessed with one-way ANOVA followed by Dunnett's test for multiple comparisons [*P* ≤ 0.05 (*), *P* ≤ 0.005 (**), *P* ≤ 0.0005 (***), *P* ≥ 0.05 (non-significant)].

## Results

### JQ1 is a PXR agonist

**Figure 2.**
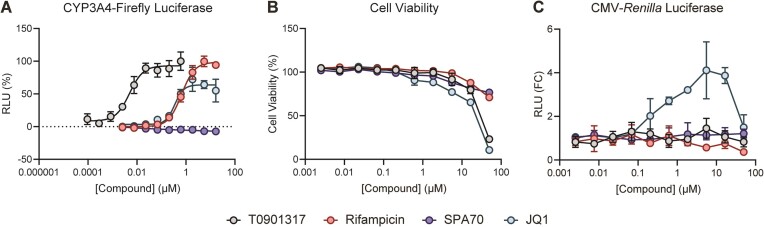
JQ1 increases reporter gene expression. (**A**) HepG2 cells were co-transfected with PXR-expressing plasmid and a plasmid encoding firefly luciferase under the control of a PXR-responsive *CYP3A4* promoter. Cells were treated with the indicated compounds for 24 h and assayed for luciferase activity. Relative light units (RLU) are plotted as percent relative to positive and negative controls (1 μM T0901317 and DMSO, respectively). (**B**) HepG2 cells were treated with the indicated compounds for 24 h and assessed for viability with the CellTiter-Glo Luminescent Cell Viability Assay. Results are plotted as percent viability relative to positive and negative controls (DMSO-treated cells and DMSO-treated media, respectively). (**C**) HepG2 cells were transfected with a plasmid encoding *Renilla* luciferase under the control of a CMV promoter. Cells were treated with the indicated compounds for 24 h and assayed for luciferase activity. Results are plotted as fold change (FC) relative to the DMSO control.

JQ1 is a CYP3A4 substrate (21), but whether it induces CYP3A4 expression is yet to be reported. Of the receptors known to regulate *CYP3A4* transcription, PXR is the main ligand-dependent *CYP3A4* regulator and is renowned for its highly promiscuous ligand binding pocket. Therefore, we assessed JQ1 for PXR activation. As controls, we used the PXR agonists T0901317 and rifampicin and the antagonist SPA70 (Figure 1) (22,30). All compounds were assayed in PXR-transfected HepG2 at 24 h for induction of a firefly luciferase reporter under the control of a PXR-responsive *CYP3A4* promoter (Figure 2A), cell viability by CellTiter-Glo (Figure 2B), and modulation of a *Renilla* luciferase reporter under the control of a CMV promoter (Figure 2C). JQ1 activated the CYP3A4-luciferase reporter with potency comparable to that of the prototypical PXR agonist rifampicin (Figure 2A) but with slightly reduced efficacy. Because JQ1 exhibited cytotoxicity at the concentrations required to activate the CYP3A4-luciferase reporter, the reduced efficacy was likely due to cytotoxicity (Figure 2B). This is supported by the observation that all test compounds reduced the CYP3A4-luciferase signal when increased to high enough concentrations (Supplementary Figure S5). Only JQ1 robustly activated *Renilla* luciferase expression from a CMV promoter, indicating general transcriptional effects independent of PXR (Figure 2C). At the highest concentrations, JQ1 also reduced CMV-*Renilla* luciferase expression, again likely due to diminished cell health (Figure 2C).

**Figure 3.**
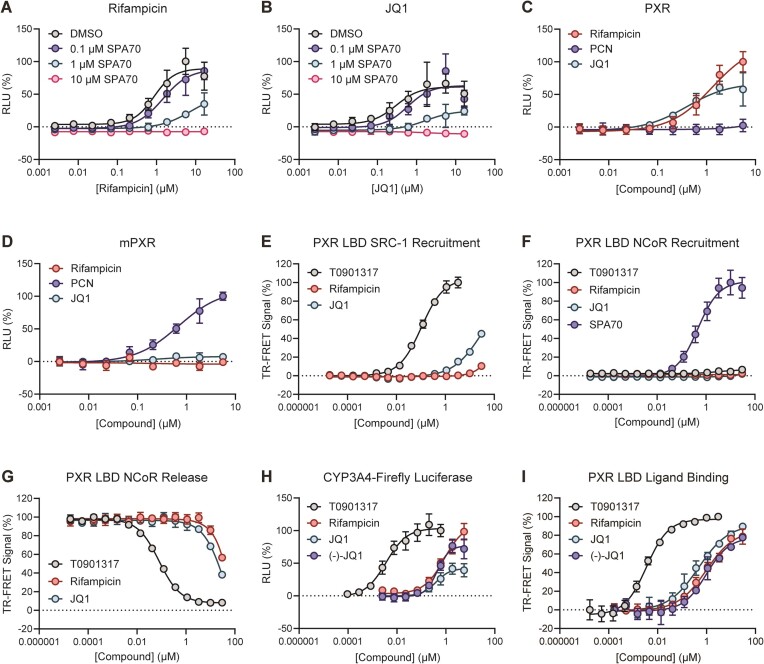
JQ1 directly binds and activates PXR. (**A, B**) HepG2 cells were co-transfected with PXR-expressing plasmid and a plasmid encoding firefly luciferase under the control of a PXR-responsive *CYP3A4* promoter. Cells were treated with (**A**) rifampicin or (**B**) JQ1 in combination with DMSO or 0.1, 1 or 10 μM SPA70 for 24 h and assayed for luciferase activity. (**C, D**) HepG2 cells were co-transfected with (**C**) PXR- or (**D**) mPXR-expressing plasmid and a plasmid encoding firefly luciferase under the control of a PXR-responsive *CYP3A4* promoter. Cells were treated with the indicated compounds for 24 h and assayed for luciferase activity. In (**D**), PCN, rather than T0901317, was used as the 100% control. (**E**) Recruitment of an SRC-1 peptide to purified PXR LBD was assessed by TR-FRET assay. The TR-FRET signal is plotted as percent relative to positive and negative controls (10 μM T0901317 and DMSO, respectively). (**F**) Recruitment of an NCoR peptide to purified PXR LBD was assessed by TR-FRET assay. The TR-FRET signal is plotted as percent relative to positive and negative controls (10 μM SPA70 and DMSO, respectively). (**G**) TR-FRET assay with purified PXR LBD and NCoR peptide was performed in the presence of 1 μM SPA70 and the indicated concentrations of agonists. The signal is plotted as percent relative to positive and negative controls (1 μM SPA70 and DMSO, respectively). Increasing concentrations of agonists displace SPA70 from the ligand binding pocket, resulting in release of NCoR peptide. (**H**) HepG2 cells were co-transfected with PXR-expressing plasmid and a plasmid encoding firefly luciferase under the control of a PXR-responsive *CYP3A4* promoter. Cells were treated with compounds for 24 h and assayed for luciferase activity. (**I**) Compound binding to purified PXR LBD was assessed by TR-FRET assay. The TR-FRET signals are plotted as percent relative to positive and negative controls (10 μM T0901317 and DMSO, respectively).

The observation that JQ1 activates both the CYP3A4-luciferase and CMV-luciferase reporters suggested that JQ1 may indiscriminately affect reporter expression, consistent with its function as a transcription modulator. JQ1-mediated enhancement of CMV-driven transcription has been previously observed and mechanistically ascribed to the transcription activator P-TEFb (47). Therefore, we conducted a series of experiments to address whether the CYP3A4-luciferase upregulation by JQ1 was due to direct PXR activation rather than other routes of transcription modulation. We first performed CYP3A4-luciferase reporter assays in the presence of the PXR antagonist SPA70 (22) to determine if JQ1-induced reporter activity can be blocked by PXR inhibition. Increasing concentrations of SPA70 blocked rifampicin-mediated PXR activation, as expected (Figure 3A). SPA70 also blocked JQ1-mediated reporter activation in a dose-dependent manner (Figure 3B), supporting that JQ1 is a PXR agonist. Because PXR ligands are generally species-specific (30), and JQ1 is used in various mouse models, we assayed JQ1 for induction of the CYP3A4-luciferase reporter in the presence of human PXR or mouse PXR (mPXR). As expected from previous characterizations, rifampicin activated human PXR but not mPXR, and PCN activated mPXR but not human PXR (Figure 3C, D). Like rifampicin, JQ1 did not activate the CYP3A4-luciferase reporter in the presence of mPXR, indicating that (i) JQ1 is not an mPXR agonist and (ii) increase of CYP3A4-luciferase signal in the presence of human PXR is due to activation of PXR by JQ1, either directly or indirectly. Because a PXR agonist is expected to induce recruitment of transcriptional coactivators (i.e. SRC-1), we assessed interaction of purified PXR LBD with a 23 amino acid SRC-1 peptide and found that JQ1 induced SRC-1 recruitment (Figure 3E and Supplementary Figure S1). Furthermore, while the antagonist SPA70 induced recruitment of corepressor NCoR (Figure 3F and Supplementary Figure S1), JQ1 inhibited SPA70-mediated NCoR recruitment (Figure 3G). Finally, PXR LBD heterodimerized with retinoid X receptor alpha (RXRα) (Supplementary Figure S2B), and the heterodimerization of PXR LBD with RXRα was not negatively affected by JQ1 and other agonists such as T0901317 and rifampicin (Supplementary Figure S2C).

To further characterize JQ1′s induction of the PXR-responsive reporter, we utilized (−)-JQ1, a JQ1 enantiomer that does not bind BET proteins and does not inhibit BET-histone interactions (Figure 1) (10). Surprisingly, we found that (−)-JQ1 activated the CYP3A4-luciferase reporter with similar potency but greater efficacy than JQ1 (Figure 3H). The enhanced signal of (−)-JQ1 compared to JQ1 may be partly attributable to decreased cytotoxicity (Supplementary Figure S6). Because (−)-JQ1 does not inhibit BET activity, the observed effect was likely due to PXR modulation and not general transcription manipulation. Consistent with this, (−)-JQ1 only marginally induced the CMV-*Renilla* luciferase reporter at high concentrations (Supplementary Figure S7). Furthermore, we identified the PGK promoter as an alternative promoter that is not positively modulated by JQ1 (Supplementary Figure S8A-C), and both JQ1 and (−)-JQ1 retained activation of the CYP3A4-luciferase reporter when PXR was expressed from a PGK promoter (Supplementary Figure S8D). To fully confirm that JQ1 and (−)-JQ1 do indeed modulate PXR by directly binding to the ligand binding pocket, we performed TR-FRET ligand binding assays with purified PXR LBD and observed that both compounds were comparable to rifampicin in binding potencies (Figure 3I), which correlates to the cellular PXR activation (Figure 3H).

### Crystal structure of PXR LBD bound to JQ1 reveals ligand malleability and a central role of the *tert*-butyl group of JQ1

**Figure 4.**
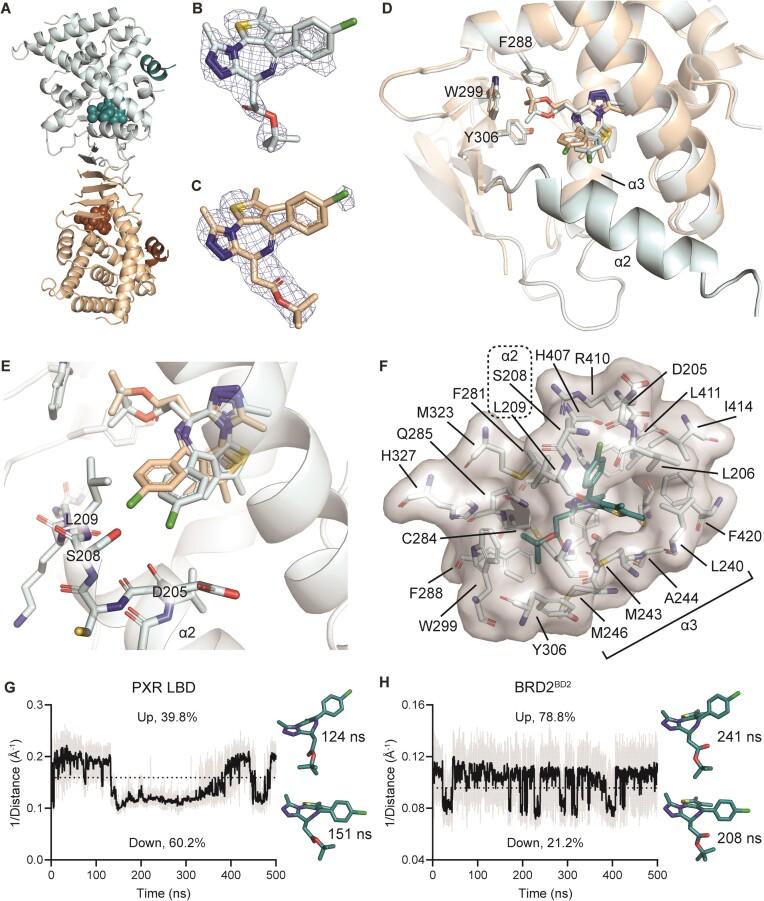
Crystal structure of JQ1-bound PXR LBD indicates ligand flexibility. (**A–F**) PXR LBD was co-crystallized with SRC-1 peptide and JQ1 to a resolution of 2.15 Å. (**A**) Two chains of PXR LBD (pale cyan and wheat colors) and two chains of SRC-1 peptide (deep teal and sand colors) were present in the asymmetric unit. (**B, C**) The 2Fo–Fc map for JQ1 in (**B**) chain A and (**C**) chain B is contoured in mesh at 1.0 rmsd and carved around JQ1 at 2 Å. (**D, E**) The two PXR LBD chains are overlaid. Alpha helix 2 (α2) was not observed in chain B. In chain B, the JQ1 chlorine clashes with the S208 and L209 residues observed in chain A, likely displacing α2. (**F**) Residues of the chain A ligand binding pocket within 5 Å of JQ1 are shown as semi-transparent surface and sticks. Carbon atoms in JQ1 are colored deep teal. (**G**) A 500 ns MD simulation was performed for PXR LBD bound to JQ1 (chain A). The distance between the JQ1 chlorine atom and the C_α_ of R410 was quantified over time and inverted so that higher values indicate the ‘up’ conformation of JQ1. The gray line shows the raw inverted distances, the black line is a 200 ps running average, and the dotted line indicates the cutoff value for ‘up’ versus ‘down.’ Examples of JQ1 ‘up’ and ‘down’ conformations are shown to the right of the plot along with the simulation times at which they occurred. (**H**) A 500 ns MD simulation was performed for BRD2^BD2^ bound to JQ1 (PDB ID 3ONI). The distance between the JQ1 chlorine atom and the C_α_ of M438 was quantified and plotted as in (**G**).

To investigate the binding mode of JQ1 to PXR LBD, we solved the PXR LBD-JQ1 co-crystal structure in the presence of coactivator SRC-1 peptide (Table 1). Consistent with previously reported structures, the asymmetric unit contained two PXR LBD (chains A and B) and two SRC-1 peptides (chains C and D) (Figure 4A). In both PXR LBD chains, the thienotriazolodiazepine core and *tert*-butyl acetate group of JQ1 were clearly resolved, but weaker density was observed for the chlorophenyl appendage (Figure 4B-C and Supplementary Figure S9). To ascertain that intact JQ1, and not a degraded molecule, was bound in the PXR LBD crystal, we conducted two tests. First, we incubated JQ1 in the crystal growth condition and used LC–MS/MS to show that the compound does not degrade over a period of eight days (Supplementary Figure S10). Importantly, our X-ray diffraction data were from a crystal that was cryoprotected after three days of growth, well within the eight-day period. Second, we collected PXR LBD-JQ1 co-crystals (*n* = 8), washed them in ten successive drops of crystal growth solution without JQ1 to remove unbound compound, and collected the washed crystals in DMSO. The ten wash solutions and the final crystal-containing DMSO were analyzed by LC–MS/MS, and intact JQ1 was identified in the washed crystals (Supplementary Figure S11). Therefore, the comparably weak chlorophenyl electron density is likely due to chemical flexibility rather than compound degradation. While the *tert*-butyl group was anchored in an aromatic cage made up of F288, W299, and Y306 that has been shown to trap a large proportion of PXR ligands (Figure 4D) (27,28,48,49), the chlorophenyl was positioned near a void and oriented toward alpha helix 2 (α2), which is highly flexible and not observed in certain previous structures (Figure 4D, E) (22,28,50). Interestingly, α2 was present in chain A but was not resolved in chain B, indicating that the close proximity of the chlorophenyl to the region displaces or destabilizes α2 (Figure 4D, E and Supplementary Figure S12).

When residues within 5 Å of JQ1 in chain A were analyzed, we observed a void between α2 (S208 and L209) and α3 (L240, M243, and A244) that may allow the chlorophenyl and additional groups to freely move (Figure 4F). Considering the observed electron density, the chlorophenyl is likely at least partially flexible. Indeed, JQ1 clearly has at least two binding poses since two different modes were observed between the two PXR LBD chains (Figure 4D, E). This plasticity and differential positioning of the *tert*-butyl acetate moiety illustrate how binding by (−)-JQ1 is possible, as there is sufficient space for either enantiomer to bind. This is unlike bromodomains, which have smaller binding pockets that constrain JQ1 to a single pose and have surrounding residues that do not allow (−)-JQ1 to bind (10). To evaluate the proposed JQ1 flexibility, we performed MD simulations of JQ1-bound PXR LBD (chain A) and the JQ1-bound second bromodomain of BRD2 (BRD2^BD2^, PDB ID 3ONI, Figure 4G, H, Supplementary Figures S13 and S14). In each simulation, we observed two major JQ1 conformations, an ‘up’ conformation and a ‘down’ conformation (Figure 4G, H, Supplementary Figure S14). The ‘up’ conformations corresponded to the positions captured in the crystal structures of both PXR LBD and BRD2^BD2^. In the ‘down’ conformations, the chlorophenyl groups were positioned downward relative to the original location. Using static reference points located above the chlorophenyl group (C_α_ of R410 in PXR LBD and C_α_ of M438 in BRD2^BD2^, Supplementary Figure S14), we quantified the residence times of the ‘up’ and ‘down’ conformations and found that the ‘up’ conformation was present for 78.8% of the BRD2^BD2^ simulation but only 39.8% of the PXR LBD simulation. Furthermore, while the ‘down’ conformation was stable and long-lived in PXR LBD, it only lasted for short durations in BRD2^BD2^, quickly returning to the ‘up’ position, indicating its instability in BRD2^BD2^. Therefore, there are likely at least two stable JQ1 binding modes in PXR LBD while there is only one stable mode in BRD2^BD2^.

### Analysis of JQ1 analogs indicates importance of the *tert*-butyl group for PXR activation

**Figure 5.**
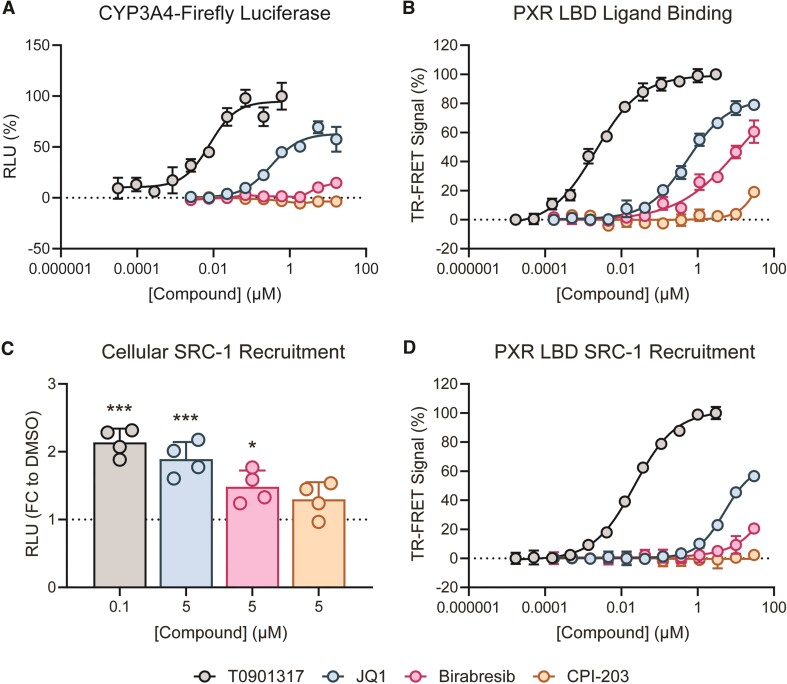
Modifications at the JQ1 *tert*-butoxy moiety alter PXR activity. (**A**) HepG2 cells were co-transfected with PXR-expressing plasmid and a plasmid encoding firefly luciferase under the control of a PXR-responsive *CYP3A4* promoter. Cells were treated with the indicated compounds for 24 h and assayed for luciferase activity. (**B**) Compound binding to purified PXR LBD was assessed by TR-FRET assay. (**C**) HepG2 cells were co-transfected with SmBiT-PXR LBD and LgBiT-tagged SRC-1 nuclear receptor interaction domain, treated with 100 nM T0901317 or 5 μM other compound for 30 min, and the interaction was measured with the Nano-Glo Live Cell Assay System. The asterisks indicate significance relative to the DMSO control using one-way ANOVA followed by Dunnett's test for multiple comparisons [*P* ≤ 0.05 (*), *P* ≤ 0.005 (**), *P* ≤ 0.0005 (***), *P* ≥ 0.05 (non-significant)]. (**D**) Recruitment of an SRC-1 peptide to purified PXR LBD was assessed by TR-FRET assay. The TR-FRET signals in (**B**) and (**D**) are plotted as percent relative to positive and negative controls (10 μM T0901317 and DMSO, respectively).

The structural results indicated that the *tert*-butyl group of JQ1 is important for binding, as it interacts with the F288/W299/Y306 aromatic cage. However, the observed electron density and MD simulations suggested that JQ1 is at least partially conformationally plastic within the PXR ligand binding pocket. Therefore, to assess the importance of the *tert*-butyl, we selected two analogs for testing that differ from JQ1 only at the *tert*-butoxy position. In birabresib, the *tert*-butoxy is replaced by a bulkier aminophenol group, and in CPI-203, the *tert*-butoxy is replaced by a simple amino group (Figure 1). When tested for induction of the PXR-responsive reporter, birabresib was a weak agonist, and CPI-203 was inactive (Figure 5A). Ligand binding assays using purified PXR LBD were consistent with this result, with binding potencies of JQ1 > birabresib > CPI-203 (Figure 5B). Interestingly, although birabresib had decreased binding potency, the compound did exhibit PXR LBD binding, indicating that birabresib's binding mode is altered compared to JQ1. However, this binding failed to translate to substantial cellular PXR activation.

Next, we studied recruitment of the SRC-1 nuclear receptor interaction domain to PXR LBD in both cellular and biochemical contexts. Mammalian two-hybrid is the typical approach to assess nuclear receptor-cofactor interactions in cells. These assays require transcription and translation of a reporter, and up to 24 h is necessary for robust signal generation. Because BET inhibitors are transcriptional modulators that can broadly affect reporter expression (Figure 2C), we used the NanoBiT system (33) with SmBiT-tagged PXR LBD and LgBiT-tagged SRC-1 nuclear receptor interaction domain to detect the interaction. NanoBiT allows signal generation within minutes rather than hours, so we were able to evaluate the BET inhibitors with negligible signal contribution from transcriptional effects. As shown in Figure 5C, T0901317 induced SRC-1 recruitment to PXR LBD, validating the assay. Of the BET inhibitors, the SRC-1 recruitment activity was JQ1 > birabresib > CPI-203, consistent with the reporter and binding results. This trend also held true when recruitment of the 23 amino acid SRC-1 peptide to purified PXR LBD was assessed (Figure 5D).

### JQ1 modulates endogenous *CYP3A4* expression in HepaRG cells by multiple mechanisms

**Figure 6.**
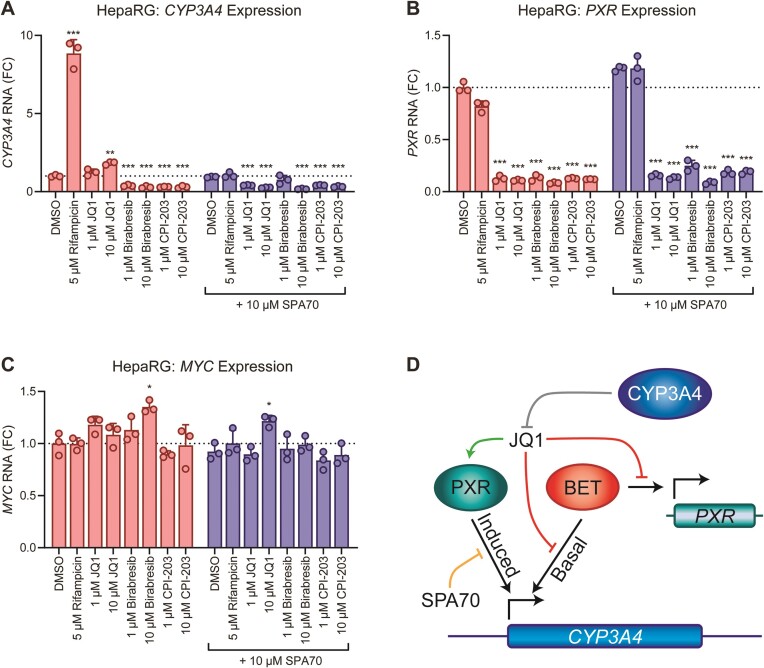
JQ1 modulates cellular *CYP3A4* expression by multiple mechanisms. (**A–C**) HepaRG cells were treated with the indicated compounds for 24 h, and RNA was extracted and subjected to RT-qPCR to measure expression of (**A**) *CYP3A4*, (**B**) *PXR* or (**C**) *MYC*. Data were normalized to *18S* RNA and represent FC relative to the DMSO control. The asterisks indicate significance relative to the corresponding group DMSO control using one-way ANOVA followed by Dunnett's test for multiple comparisons [*P* ≤ 0.05 (*), *P* ≤ 0.005 (**), *P* ≤ 0.0005 (***), *P* ≥ 0.05 (non-significant)]. (**D**) The schematic illustrates the multiple effects of BET inhibitors on *CYP3A4* expression, with a focus on JQ1. JQ1 binds and activates PXR (green arrow), leading to increased *CYP3A4* expression. However, all BET inhibitors reduce basal *CYP3A4* expression (red blunt line), suggesting BET proteins contribute to basal *CYP3A4* transcription. Additionally, BET inhibitors decrease *PXR* expression (red blunt line), further diminishing JQ1-induced *CYP3A4* upregulation through PXR. Lastly, CYP3A4 metabolizes JQ1 (gray blunt line), leading to diminished biological activity. SPA70 antagonizes binding of PXR agonist, such as JQ1 (orange blunt arrow).

To evaluate how JQ1 affects PXR-dependent transcriptional readouts in a more physiologically relevant context, we assessed induction of endogenous *CYP3A4* mRNA by endogenous PXR in the HepaRG cell model. HepaRG cells are hepatic cells derived from a human hepatic progenitor cell line that retain many features of primary human hepatocytes, including the expression of PXR and CYPs (e.g. CYP3A4) (45,51–53). In this model, rifampicin robustly induced expression of *CYP3A4* mRNA, and co-treatment with SPA70 blocked such induction (Figure 6A). JQ1 treatment modestly induced *CYP3A4* mRNA, and the induction was blocked by SPA70, suggesting that the induction was indeed through PXR. Interestingly, though, not only did SPA70 block JQ1-mediated *CYP3A4* induction, but *CYP3A4* expression was largely diminished in cells co-treated with JQ1 and SPA70. Furthermore, both birabresib and CPI-203, which do not activate PXR (Figure 5), reduced *CYP3A4* RNA level in the presence and absence of SPA70. These observations suggest that in HepaRG cells, BET proteins contribute to the basal transcription of the *CYP3A4* gene, and inhibition of BET proteins reduces *CYP3A4* transcription independently of PXR. In support of this, (−)-JQ1 more robustly induced *CYP3A4* expression than JQ1 without reducing *CYP3A4* in the presence of SPA70 (Supplementary Figure S15A). Furthermore, JQ1, birabresib, and CPI-203 also greatly reduced *PXR* RNA (Figure 6B), which would in turn result in decreased PXR protein and induction capacity. Likewise, the induction capacity of (−)-JQ1 seemed to be restricted at 10 μM due to reduction of *PXR* RNA, but the observed effect was much less than that observed with the BET inhibitors (Supplementary Figure S15B, C). *MYC* is a known BET target gene and was used as a control (54). The compounds did not affect *MYC* expression (Figure 6C), consistent with previous reports using JQ1 in other liver cell models (18). Lack of *MYC* sensitivity to BET inhibition has been shown in additional cell models, such as fibroblasts (55), and it has been noted that the *MYC* locus has cell type-specific enhancer configurations that might be differentially sensitive to BET inhibition (56).

## Discussion

We have found that JQ1 is a potent and efficacious PXR agonist, with activity comparable to that of the prototypical PXR agonist, rifampicin. JQ1 exhibits the expected features of PXR agonism, including direct binding to PXR LBD, induced association of PXR LBD with the coactivator SRC-1, and loss of PXR activation in the presence of the PXR antagonist SPA70. JQ1 does not activate mPXR, however, so PXR activation in mouse models is unlikely to occur, suggesting that species difference, informed by mechanistic studies, needs to be carefully considered when extrapolating animal data to human applications. Importantly, we determined that the *tert*-butyl moiety of JQ1 is a main chemical determinant of PXR activation and have identified a possible method to mitigate the PXR activity of JQ1 analogs. However, our observation that birabresib retains a certain level of PXR binding suggests that related compounds may bind to PXR in unexpected orientations that were not captured in our PXR LBD-JQ1 co-crystal structure. The flexibility observed in the PXR LBD-JQ1 co-crystal structure contrasts with the constrained binding poses in bromodomain-JQ1 structures (10), highlighting the structural features that allow PXR to bind diverse molecules.

JQ1 is a highly successful probe compound that opened the door to BET chemical biology. The field progressed rapidly from JQ1 discovery to clinical trials of BET inhibitors in a range of diseases, but the full biological impacts of BET inhibition are unknown. BET proteins are ubiquitously expressed across human cell types, but their roles vary drastically among cell types and pathologies (57). Therefore, careful and thorough evaluation of BET biology and inhibitors must be conducted for various cell and tissue systems. This proposal is supported by the previous observation that JQ1 can promote prostate cancer invasion independently of BET proteins by directly inhibiting the FOXA1 transcription factor (58). As BET inhibitors are orally bioavailable treatments, systemic effects must be considered, including metabolic liabilities. However, because the chemicals block the activities of major transcriptional modulators, standard metabolic readouts may be insufficient to predict potential adverse effects or drug-drug interactions. Indeed, we show here that JQ1, birabresib, and CPI-203 affect *CYP3A4* expression in the widely used HepaRG model in manners that are both dependent on and independent of the known drug metabolism regulator PXR. Thus, significant metabolic events may occur even with new generation BET inhibitors that are not based on the JQ1 scaffold (59).

In summary, we report here four important findings: (i) a non-BET target of JQ1, (ii) a mechanism of physiological JQ1 instability, (iii) a biological function of (−)-JQ1 and (iv) a BET-dependent regulatory pathway for transcription of drug metabolism genes. These findings provide evidence that transcription modulators impact metabolism pathways independently of canonical regulators such as PXR and reinforce the importance of systemic evaluation of transcriptional modulators. Our results suggest that liver-specific impacts of BET inhibition are varied and complex. While JQ1 robustly activated transcription from the PXR-dependent *CYP3A4* promoter in HepG2 reporter assays, *CYP3A4* induction in the HepaRG model seemed to be countered by the suppression of *CYP3A4* transcription by BET inhibition. BET inhibitor-mediated loss of *CYP3A4* expression could potentially be a serendipitous effect since CYP3A4 is responsible for the majority of clinical drug metabolism events (20). However, if BET inhibitors were to be used in combination with other drugs, reduction of cellular CYP3A4 may lead to drug-drug interactions. For example, cotreating a patient with drug A (a BET inhibitor) in addition to drug B (a CYP3A4 substrate) would lead to enhanced plasma concentration of drug B, potentially causing adverse effects, and the dosage of drug B would need to be adjusted accordingly. More thorough investigation is necessary to fully understand the physiological ramifications, especially considering that orally administered drugs reach high concentrations in detoxification tissues, such as liver. Appropriately dissecting the full biological functions of BET inhibitors and other transcription modulators is imperative to obtain safe and effective therapeutics.

## Supplementary Material

gkad1175_supplemental_file

## Data Availability

The data underlying this article are available in the Protein Data Bank under Accession ID 8F5Y.
