# Supplementary material for: A bromodomain-independent mechanism of gene regulation by the BET inhibitor JQ1: direct activation of nuclear receptor PXR
Source: Nucleic Acids Res. 2023 Dec 12;52(4):1661–76. doi: 10.1093/nar/gkad1175 (PMC10899790; doi:10.1093/nar/gkad1175)
Supplement: gkad1175_supplemental_file [file gkad1175_supplemental_file.pdf]

SUPPLEMENTAL INFORMATION

A Bromodomain-Independent Mechanism of Gene Regulation by the BET Inhibitor JQ1: Direct Activation of Nuclear Receptor PXR

Andrew D. Huber[1], Shyaron Poudel[1], Jing Wu[1], Darcie J. Miller[2], Wenwei Lin[1], Lei Yang[1], Monicah N. Bwayi[1], Mary Ashley Rimmer[1], Rebecca R. Florke Gee[1,3], Jayaraman Seetharaman[2], Sergio C. Chai[1], and Taosheng Chen[1,*]

[1]Department of Chemical Biology and Therapeutics, St. Jude Children’s Research Hospital, 262 Danny Thomas Place, Memphis, TN, 38105, USA

[2]Department of Structural Biology, St. Jude Children's Research Hospital, 262 Danny Thomas Place, Memphis, TN, 38105, USA

[3]Graduate School of Biomedical Sciences, St. Jude Children's Research Hospital, 262 Danny Thomas Place, Memphis, TN, 38105, USA

*Corresponding author; address: 262 Danny Thomas Place, MS 1000, Memphis, TN 38105-3678; email address: taosheng.chen@stjude.org; phone: (901) 595-5937

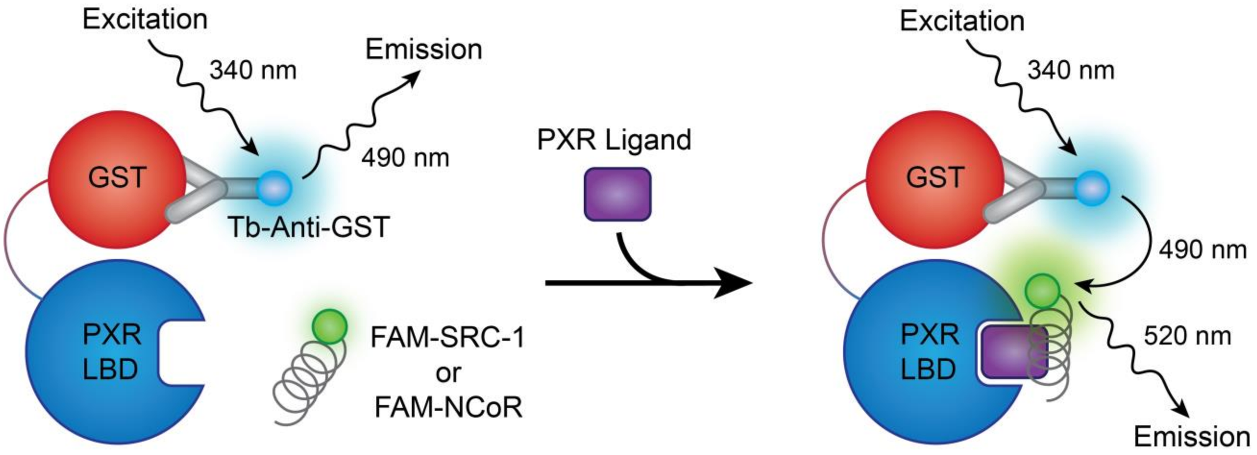

$$\text{Equation 2: Signal} = \frac{\text{RFU at 520 nm}}{\text{RFU at 490 nm}}$$

$$\text{Equation 3, SRC-1: TR-FRET Signal (\%)} = 100 \times \frac{\text{Signal}_{\text{Chemical}} - \text{Signal}_{\text{DMSO}}}{\text{Signal}_{\text{T0901317}} - \text{Signal}_{\text{DMSO}}}$$

$$\text{Equation 3, NCoR: TR-FRET Signal (\%)} = 100 \times \frac{\text{Signal}_{\text{Chemical}} - \text{Signal}_{\text{DMSO}}}{\text{Signal}_{\text{SPA70}} - \text{Signal}_{\text{DMSO}}}$$

Figure S1. TR-FRET PXR LBD SRC-1 and NCoR recruitment assays. The TR-FRET PXR LBD SRC-1 and NCoR recruitment assays measure PXR LBD interaction with a fluorescently labeled SRC-1 (FAM-SRC-1) or NCoR (FAM-NCoR) peptide. RFU, relative fluorescence units.

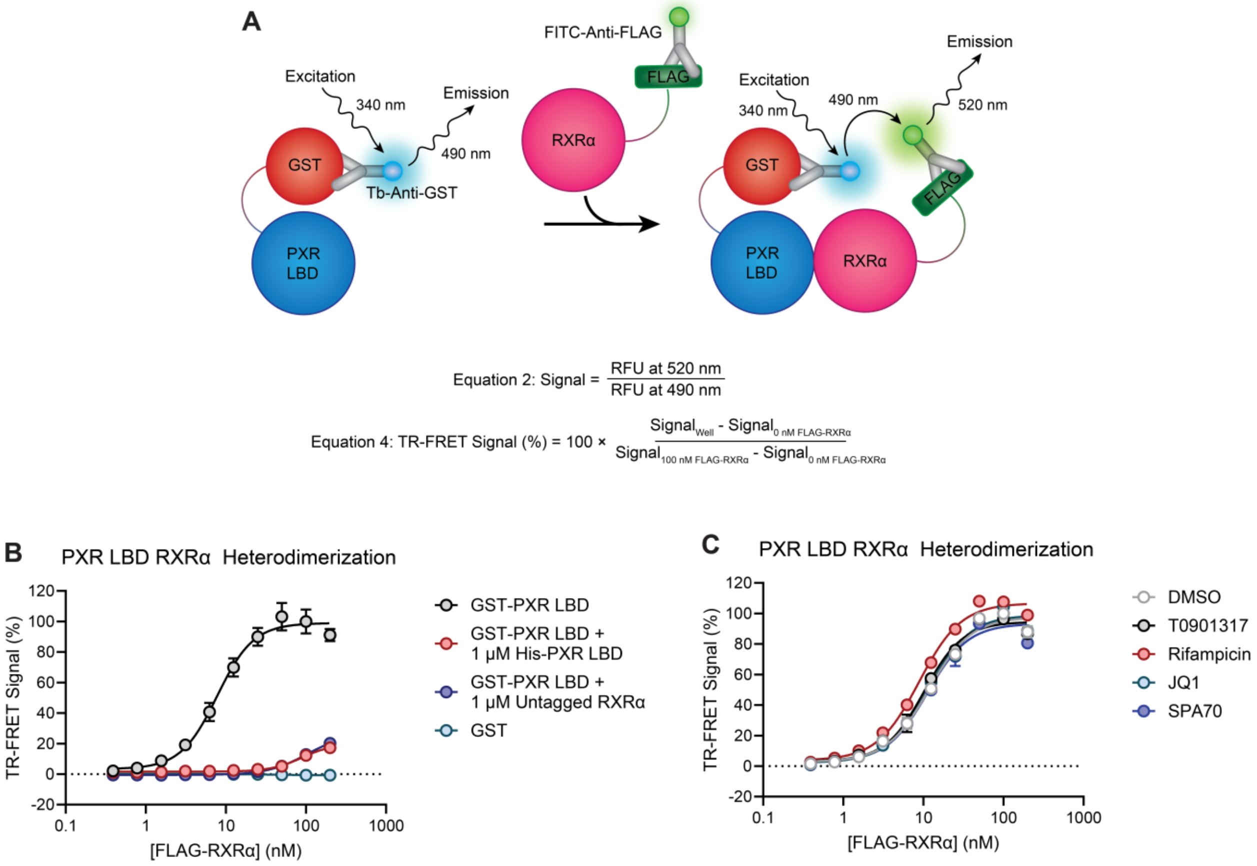

Figure S2. TR-FRET PXR LBD RXRα heterodimerization assay. (**A**) The TR-FRET PXR LBD RXRα heterodimerization assay measures PXR LBD interaction with full-length RXRα. (**B**) Increasing concentrations of FLAG-RXRα were added to wells containing 3 nM GST-PXR LBD or unfused GST protein. The interaction of GST-PXR LBD with FLAG-RXRα was diminished in the presence of His-PXR LBD or untagged RXRα, indicating that the complex between GST-PXR LBD and FLAG-RXRα can be disrupted. There was no signal observed with unfused GST protein, indicating that signal generation is specific to PXR LBD-RXRα interaction. (**C**) Increasing concentrations of FLAG-RXRα were added to wells containing 3 nM GST-PXR LBD in the presence of 0.5% DMSO or 10 μM of the indicated ligand. Ligands did not significantly alter the efficiency of PXR LBD-RXRα heterodimerization.

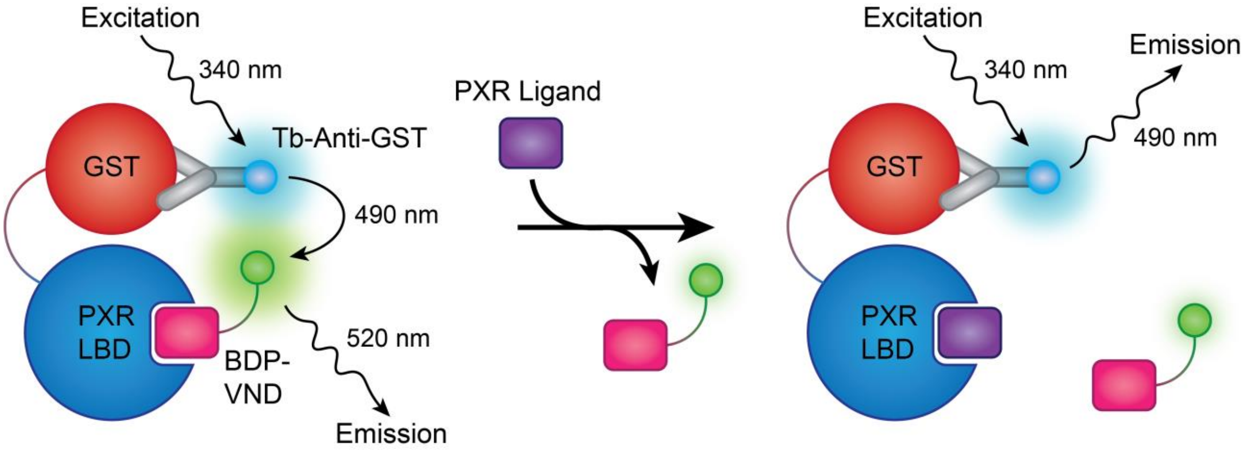

$$\text{Equation 2: Signal} = \frac{\text{RFU at 520 nm}}{\text{RFU at 490 nm}}$$

$$\text{Equation 5: TR-FRET Signal (\%)} = 100 \times \left(1 - \frac{\text{Signal}_{\text{Chemical}} - \text{Signal}_{\text{T0901317}}}{\text{Signal}_{\text{DMSO}} - \text{Signal}_{\text{T0901317}}}\right)$$

Figure S3. TR-FRET PXR LBD ligand binding assay. The TR-FRET PXR LBD ligand binding assay measures binding of PXR ligands by competition with the fluorescently labeled ligand BODIPY FL vindoline (BDP-VND).

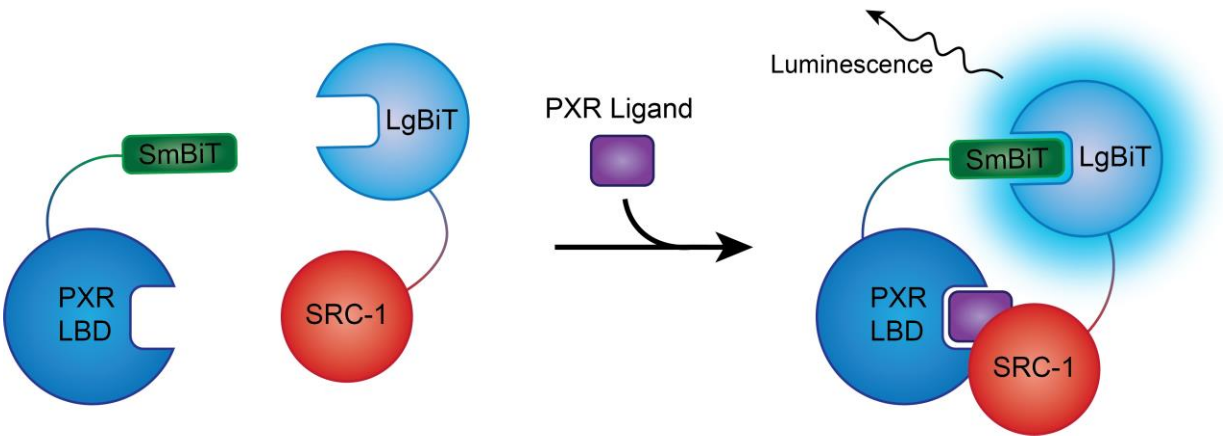

Figure S4. Cellular PXR LBD SRC-1 recruitment assay. The NanoBiT system was used to assess the interaction of coactivator SRC-1 with PXR LBD in HepG2 cells.

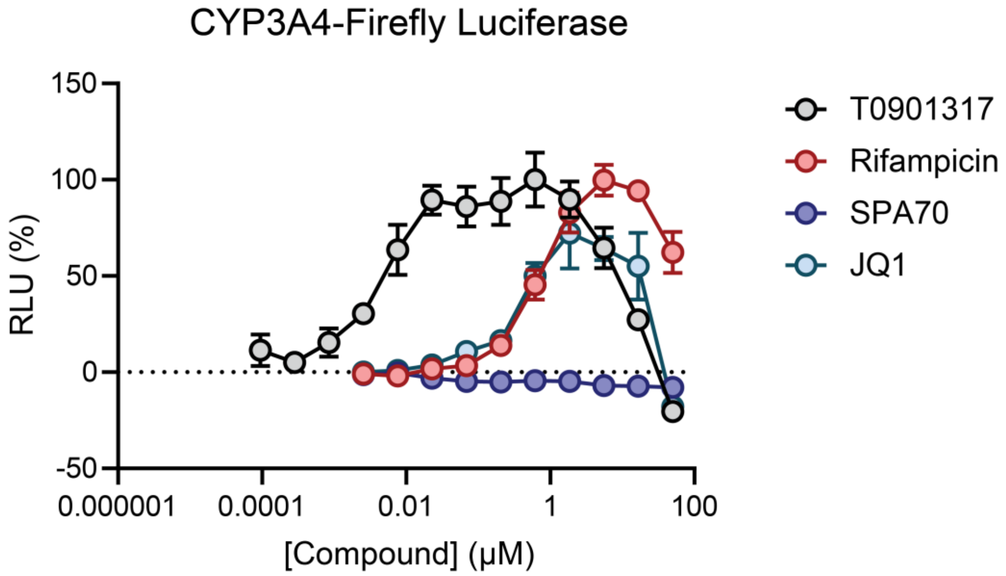

Figure S5. Cytotoxic concentrations of compounds reduce CYP3A4-luciferase reporter signal. HepG2 cells were co-transfected with PXR-expressing plasmid and a plasmid encoding firefly luciferase under the control of a PXR-responsive *CYP3A4* promoter. Cells were treated with the indicated compounds for 24 h and assayed for luciferase activity. Relative light units (RLU) are plotted as percent relative to positive and negative controls (1 μM T0901317 and DMSO, respectively). The plot shows the extended full curves of the data from Figure 2A.

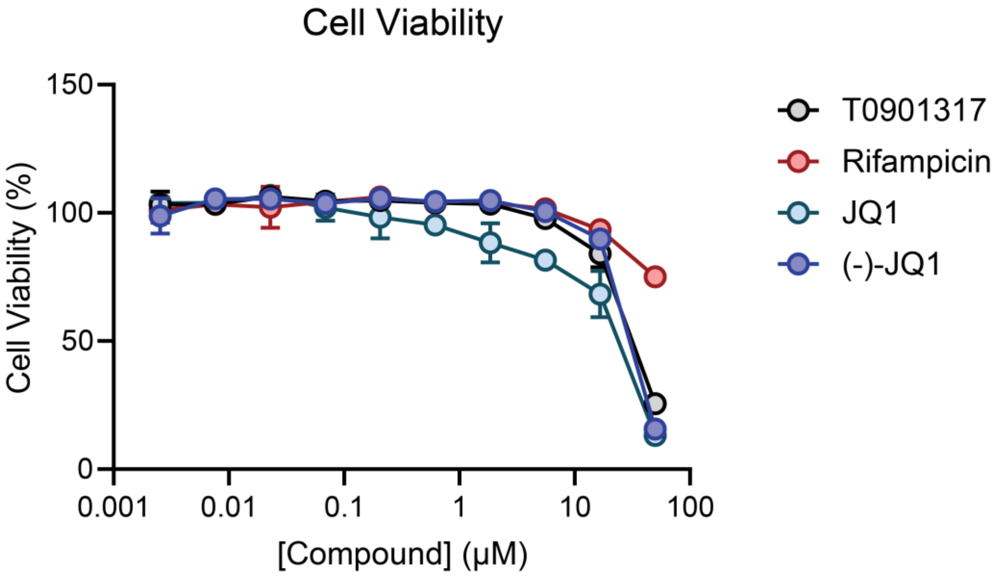

Figure S6. (-)-JQ1 is less cytotoxic than JQ1. HepG2 cells were treated with the indicated compounds for 24 h and assessed for viability with the CellTiter-Glo Luminescent Cell Viability Assay. Results are plotted as percent viability relative to the DMSO control.

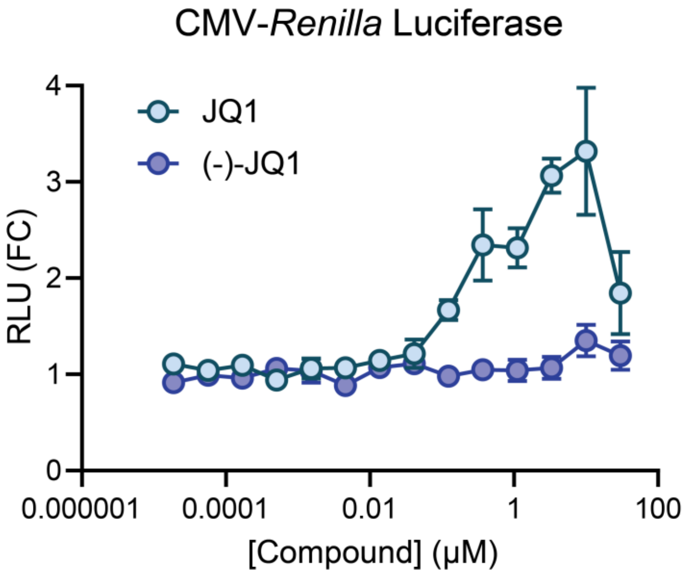

Figure S7. (-)-JQ1 does not activate the *CMV* promoter. HepG2 cells were transfected with a plasmid encoding *Renilla* luciferase under the control of a *CMV* promoter. Cells were treated with the indicated compounds for 24 h and assayed for luciferase activity. Results are plotted as fold change (FC) relative to the DMSO control.

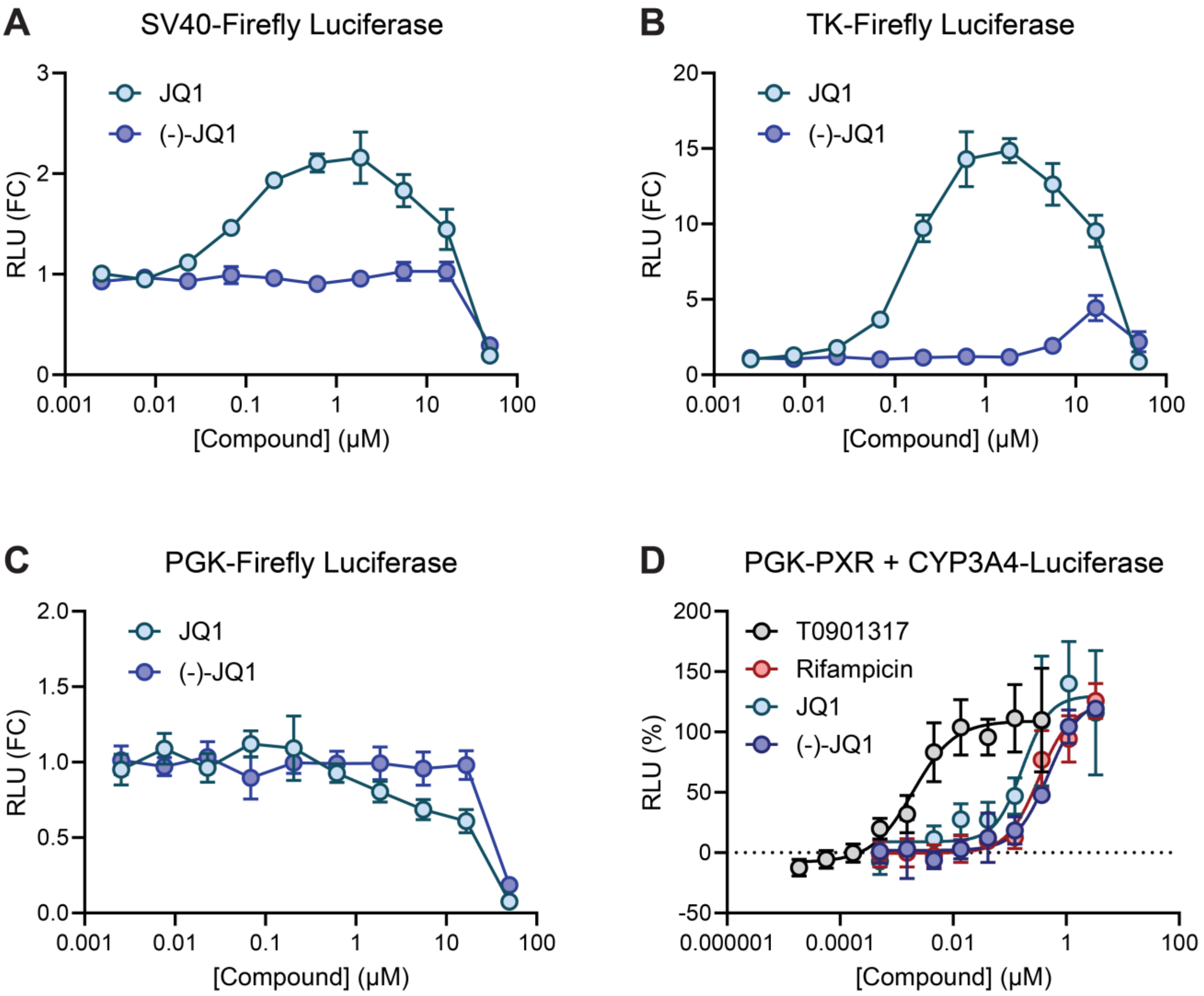

Figure S8. JQ1 does not activate the *PGK* promoter. (**A**-**C**) HepG2 cells were transfected with a plasmid encoding firefly luciferase under the control of the (**A**) *SV40*, (**B**) *TK*, or (**C**) *PGK* promoter. Cells were treated with the indicated compounds for 24 h and assayed for luciferase activity. Results are plotted as fold change (FC) relative to the DMSO control. (**D**) HepG2 cells were co-transfected with a plasmid encoding firefly luciferase under the control of a PXR-responsive *CYP3A4* promoter and a plasmid encoding PXR expressed from the *PGK* promoter. Cells were treated with the indicated compounds for 24 h and assayed for luciferase activity. Relative light units (RLU) are plotted as percent relative to positive and negative controls (1 μM T0901317 and DMSO, respectively).

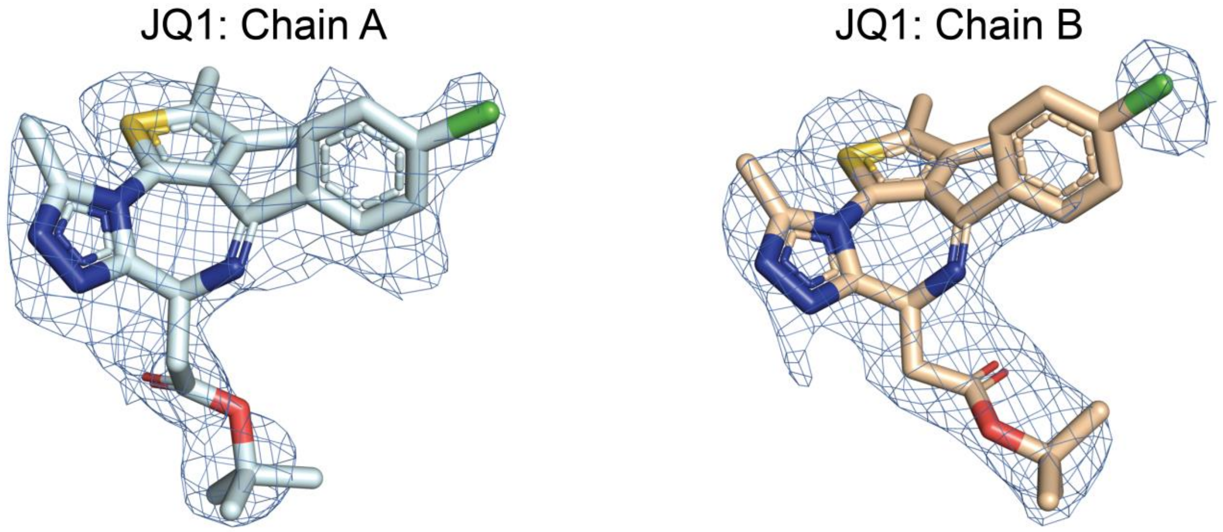

Figure S9. Electron density for JQ1. The 2Fo–Fc map for JQ1 in chains A and B is contoured in mesh at 0.7 rmsd and carved around JQ1 at 2 Å.

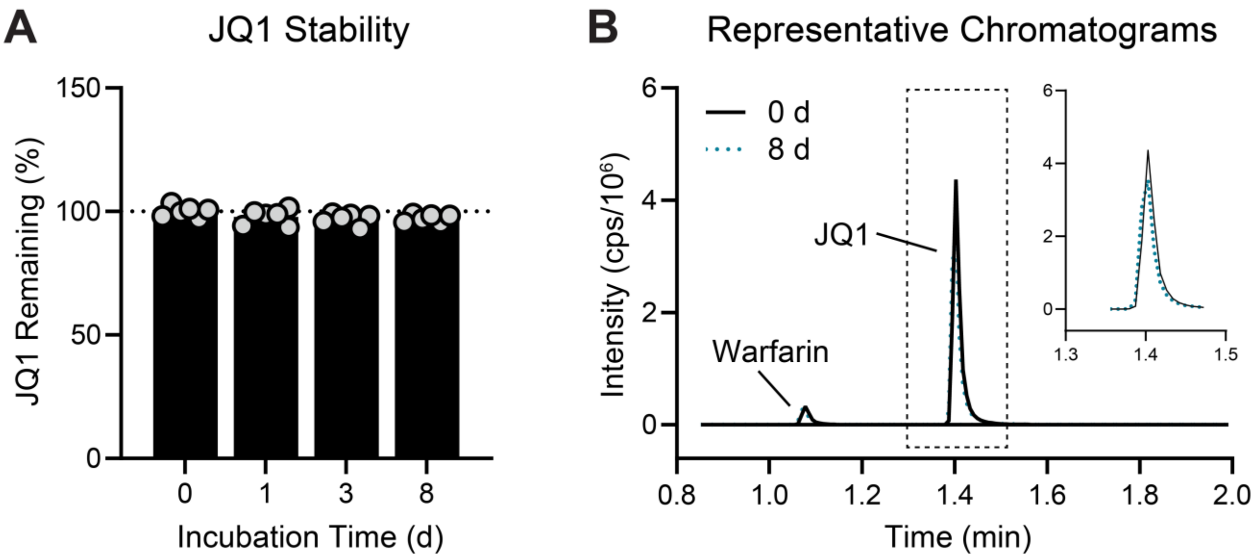

Figure S10. JQ1 is stable in the crystallographic condition. (**A**) JQ1 was incubated at 20°C in crystallization solution for the indicated times and analyzed by LC-MS/MS. The calculated intact JQ1 concentration remaining at each time is plotted as percent relative to the 0-day samples. The crystallization solution contained 25 mM imidazole, 5% isopropanol, 10 mM Tris, 100 mM NaCl, 2.5% (v/v) glycerol, 2.5 mM DTT, and 1.25 mM EDTA and had a final pH of 7.4. This composition matched the final composition of a crystallization drop containing a 1:1 ratio of protein to reservoir solution. (**B**) Representative UPLC chromatograms for 0-day and 8-day samples. The inset shows a zoomed in view of the JQ1 peaks (cps, counts per second).

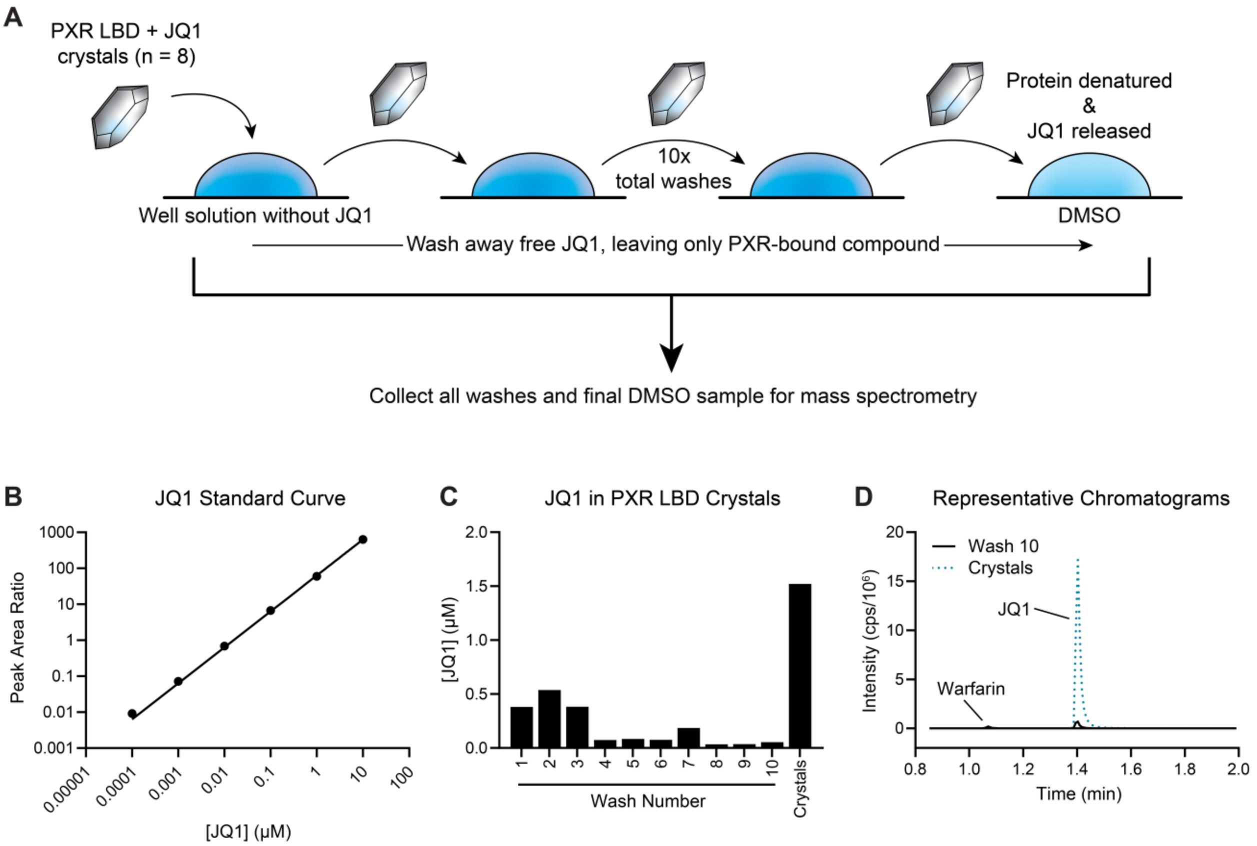

Figure S11. Intact JQ1 is bound in the PXR LBD crystals. (**A**) PXR LBD-JQ1 co-crystals were grown, collected (n = 8), and washed in ten successive drops (5 µL each) of crystallization solution to remove unbound JQ1. The final washed crystals were combined into 5 µL DMSO. The ten wash solutions and the final DMSO-dissolved crystal solution were analyzed by LC-MS/MS. The crystallization solution contained 25 mM imidazole, 5% isopropanol, 10 mM Tris, 100 mM NaCl, 2.5% (v/v) glycerol, 2.5 mM DTT, and 1.25 mM EDTA and had a final pH of 7.4. This composition matched the final composition of a crystallization drop containing a 1:1 ratio of protein to reservoir solution. (**B**) LC-MS/MS standard curve of JQ1 concentration. (**C**) The calculated JQ1 concentration in each of the ten wash steps and the final dissolved crystals is plotted. The concentration of JQ1 decreases with washing and dramatically increases in the DMSO-dissolved crystal sample. (**D**) Representative UPLC chromatograms for the tenth wash and DMSO-dissolved crystals.

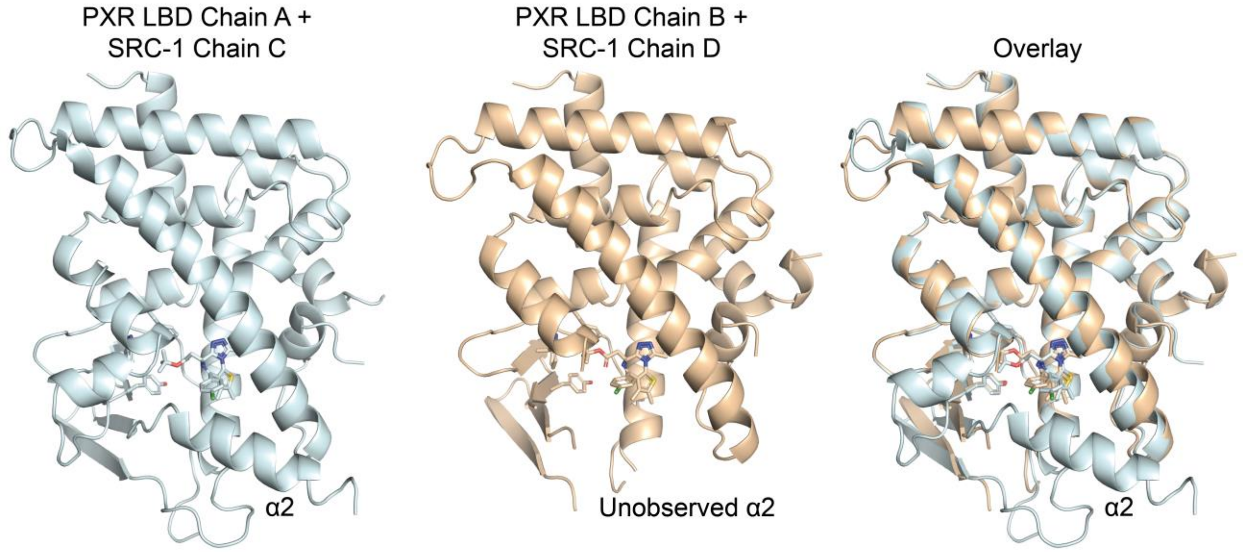

Figure S12. Alpha helix 2 (α2) is observed in PXR LBD chain A but not PXR LBD chain B. The two PXR LBD chains in the asymmetric unit are shown, along with the corresponding SRC-1 chains.

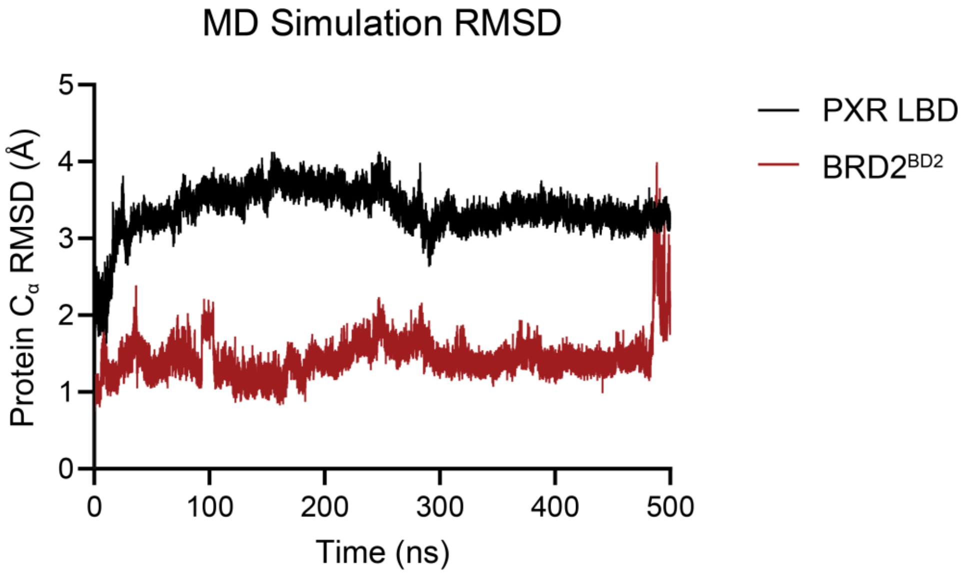

Figure S13. MD simulations were equilibrated. 500 ns MD simulations were performed for PXR LBD bound to JQ1 (chain A) and BRD2^{BD2} bound to JQ1 (PDB ID 3ONI). The root mean square deviation (RMSD) of the protein C_{α} atoms is plotted for each frame (every 10 ps). The N- and C-termini of BRD2^{BD2} underwent simultaneous movements at the end of the simulation that resulted in an RMSD spike; however, the termini quickly returned to the equilibrated state.

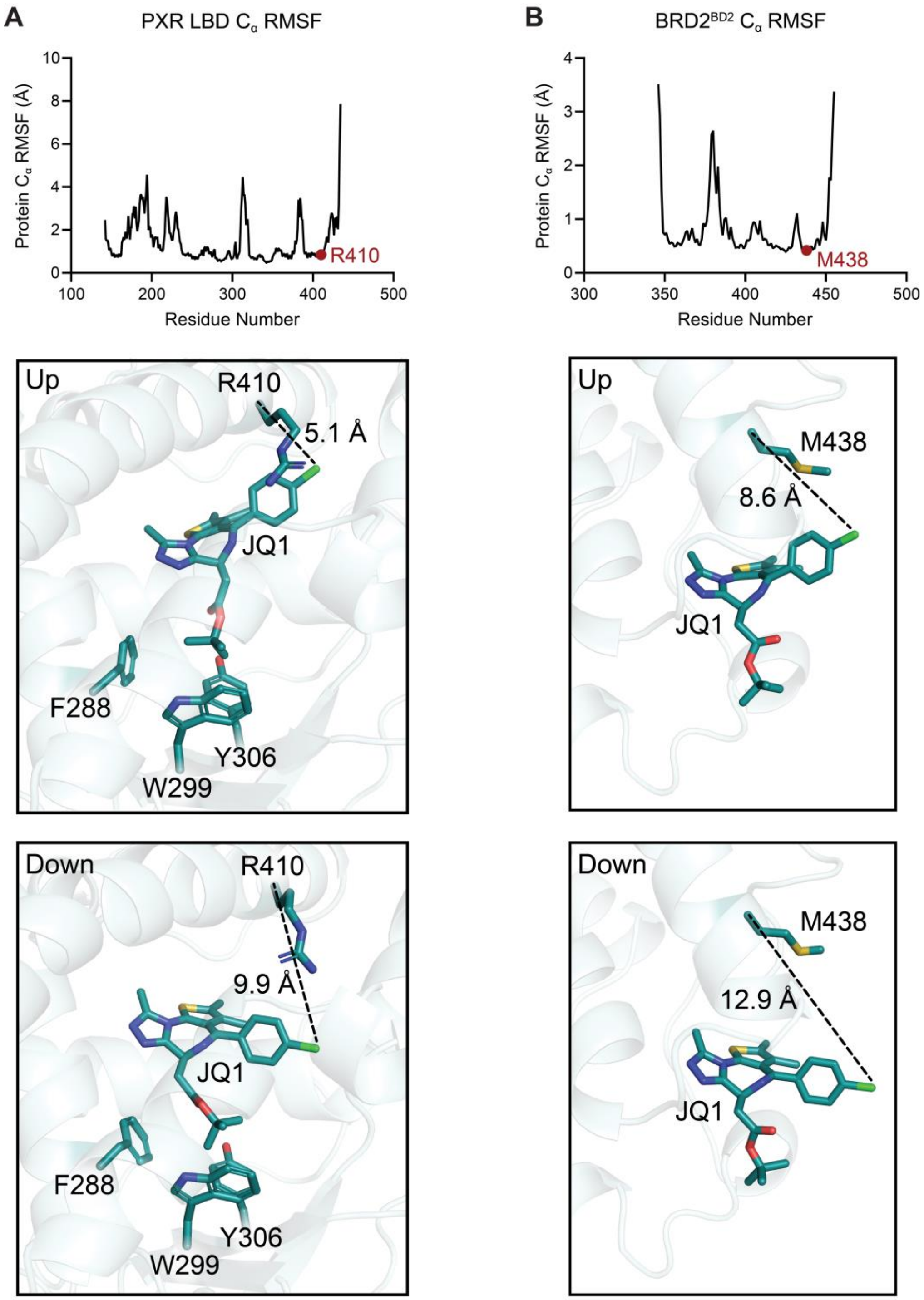

Figure S14. JQ1 adopts two major poses in MD simulations. The root mean square fluctuation (RMSF) of the protein C_α atoms is plotted for each residue. The C_α atoms of R410 in PXR LBD and M438 in $BRD2^{BD2}$ were chosen to quantify the "up" and "down" JQ1 conformations because of their low RMSF and their positions directly above the JQ1 chlorophenyl group.

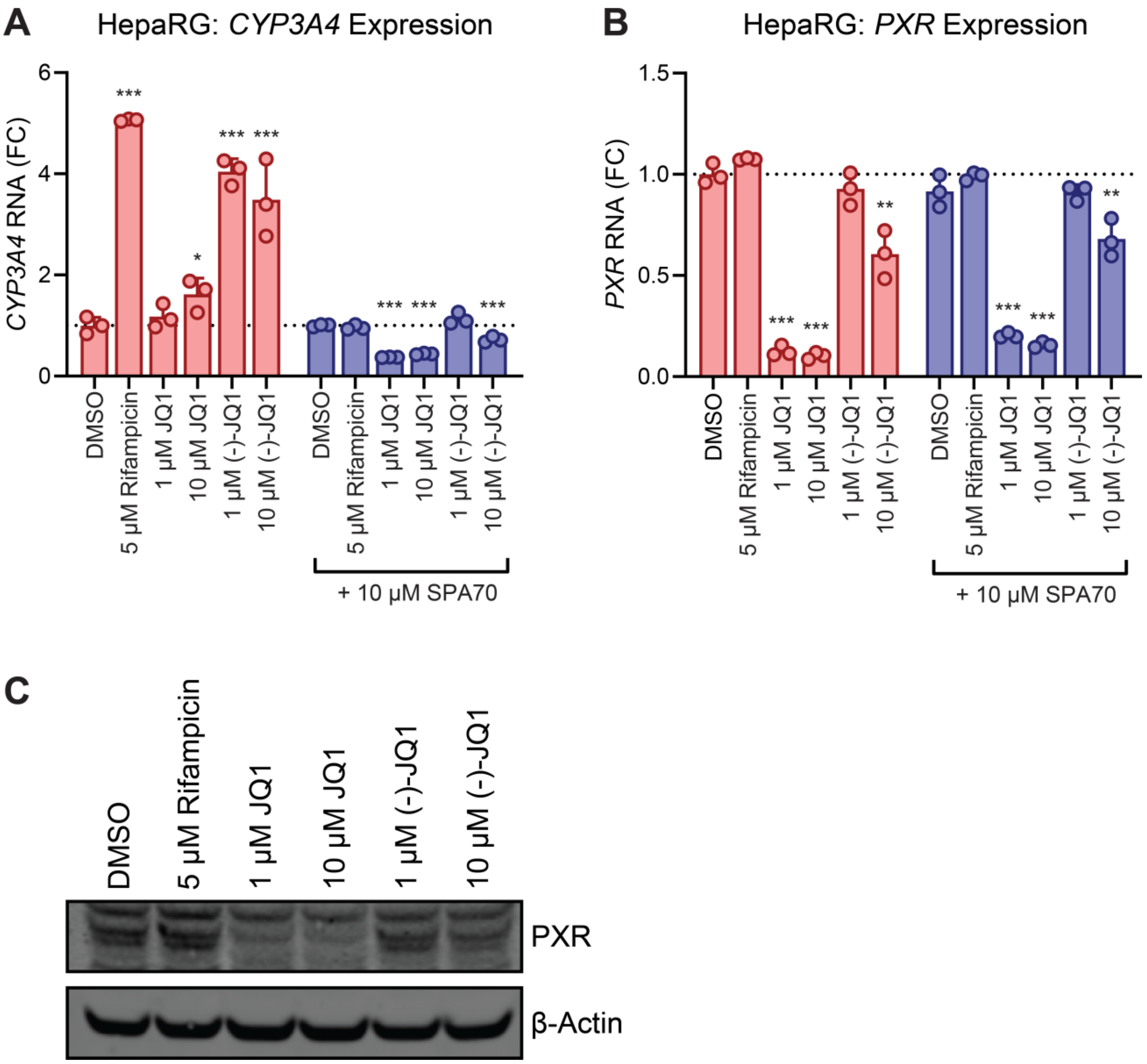

Figure S15. (-)-JQ1 induces endogenous *CYP3A4* expression more robustly than JQ1. (**A-B**) HepaRG cells were treated with the indicated compounds for 24 h, and RNA was extracted and subjected to RT-qPCR to measure expression of (**A**) *CYP3A4* or (**B**) *PXR*. Data were normalized to *18S* RNA and represent fold change (FC) relative to the DMSO control. (**C**) HepaRG cells were treated with the indicated compounds for 24 h, and protein was extracted and subjected to western blot with PXR or β-actin antibody.
